# PARP inhibition with olaparib and talazoparib for HER2-negative advanced breast cancer—Results from the prospective PRAEGNANT registry

**DOI:** 10.1038/s41523-026-00947-8

**Published:** 2026-04-11

**Authors:** Manuel Hörner, Andreas Hartkopf, Nelson John, Philipp Ziegler, Lothar Häberle, Sabrina Uhrig, Chloë Goossens, Niklas Amann, Jan-Philipp Cieslik, Lara M. Tretschock, Dominik Dannehl, Thomas M. Deutsch, Moritz Dimpfl, Max Ehlert, Kathleen Eichstädt, Alexander Englisch, Melitta B. Köpke, Annika Krückel, Theresa Link, Annika Müller, Kristin Reinhardt, Jonas Roth, Henning Schäffler, Lea Sych, Nikolas Tauber, Christian M. Tegeler, Catharina Wichmann, Maggie Banys-Paluchowski, Henriette Princk, Achim Rody, Sara Y. Brucker, Nina Ditsch, Johannes Ettl, Tanja Fehm, Carolin C. Hack, Peyman Hadji, Alexander Hein, Wolfgang W. Janni, Hans-Christian Kolberg, Diana Lüftner, Michael P. Lux, Volkmar Müller, Florin-Andrei Taran, Hans Tesch, Diethelm Wallwiener, Frederik Marmé, Stephan Seitz, Erik Belleville, Laura L. Michel, Markus Wallwiener, Peter A. Fasching, Andreas Schneeweiss, Christian Maurer

**Affiliations:** 1 Department of Gynecology and Obstetrics, University Hospital Erlangen, Comprehensive Cancer Center Erlangen-EMN (CCC ER-EMN), Friedrich-Alexander-Universität Erlangen-Nürnberg (FAU), Erlangen, Germany; 2 Department of Women’s Health, Tuebingen University, Tuebingen, Germany; 3 Biostatistics Unit, Erlangen University Hospital, Department of Gynecology and Obstetrics, University Hospital Erlangen, Comprehensive Cancer Center Erlangen-EMN (CCC ER-EMN), Friedrich-Alexander-Universität Erlangen-Nürnberg (FAU), Erlangen, Germany; 4 Department of Gynecology and Obstetrics, Center of Integrated Oncology ABCD, Medical Faculty, Heinrich Heine University and University Hospital Düsseldorf, Düsseldorf, Germany; 5 Department of Obstetrics and Gynecology, University Hospital Heidelberg, Heidelberg, Germany; 6 Department of Gynecology and Gynecologic Oncology, Center for Integrated Oncology Aachen Bonn Cologne Düsseldorf, Medical Faculty and University Clinic of Cologne, University of Cologne, Cologne, Germany; 7 Department of Gynecology and Obstetrics, University Medical Center Mannheim, Medical Faculty Mannheim, Heidelberg University, Mannheim, Germany; 8 University Medical Center Hamburg-Eppendorf, Hamburg, Germany; 9 Department of Gynecology, University Hospital Halle, Halle, Germany; 10 Department of Obstetrics and Gynaecology, University Hospital Augsburg, Augsburg, Germany; 11 Department of Gynecology and Obstetrics, Medical Faculty and University Hospital Carl Gustav Carus, Technische Universität Dresden, Dresden, Germany; 12 Department of Obstetrics and Gynecology, Caritas Hospital St. Josef, University Medical Center Regensburg,, University of Regensburg, Regensburg, Germany; 13 Department of Gynecology and Obstetrics, University Hospital Ulm, Ulm, Germany; 14 Department of Medical Oncology, National Center for Tumor Diseases (NCT), University Hospital Heidelberg, Heidelberg, Germany; 15 Department of Gynecology and Obstetrics, University Hospital Schleswig-Holstein, Campus Lübeck, Luebeck, Germany; 16 Department of Obstetrics and Gynecology, Klinikum Rechts Der Isar, Technical University of Munich, Munich, Germany; 17 Cancer Center Kempten/Allgäu (CCKA), Klinikum Kempten, Kempten, Germany; 18 Frankfurt Center for Bone Health and Endocrinology, Frankfurt Am Main, Germany; 19 Department of Gynecology, Klinikum Esslingen GmbH, Esslingen, Germany; 20 Department of Gynecology and Obstetrics, Marienhospital Bottrop, Bottrop, Germany; 21 Immanuel Hospital Märkische Schweiz & Immanuel Campus Rüdersdorf, Medical University of Brandenburg Theodor-Fontane, Rüdersdorf bei Berlin, Germany; 22 Department of Gynecology and Obstetrics, Frauenklinik St. Louise, Paderborn, St. Josefs-Krankenhaus, Salzkotten, Vincenz Kliniken GmbH, Paderborn, Germany; 23 Oncology Practice, Bethanien Hospital, Frankfurt am Main, Germany; 24 ClinSol GmbH & Co. KG, Würzburg, Germany; 25 German Cancer Research Center (DKFZ), Heidelberg, Germany; 26 Department of Gynecology and Gynecologic Oncology and Department I of Internal Medicine, Center for Integrated Oncology Aachen Bonn Cologne Duesseldorf, Faculty of Medicine and University Hospital Cologne, University of Cologne, Cologne, Germany

**Keywords:** Cancer, Drug discovery, Oncology

## Abstract

Germline *BRCA1* and *BRCA2* mutations enable targeted therapies in human epidermal growth factor receptor 2 (HER2)-negative advanced breast cancer (ABC). The two poly (adenosine diphosphate-ribose) polymerase (PARP) inhibitors olaparib and talazoparib were introduced into clinical practice in 2018. Limited evidence about their routine clinical use highlights the importance of this analysis. We provide a real-world analysis for PARP-inhibitor use in ABC patients treated within the prospective German PRAEGNANT registry (NCT02338167). 152 patients with ABC receiving a PARP-inhibitor were included. Real-world progression-free survival (rwPFS) and real-world overall survival (rwOS) were calculated for all patients using the Kaplan–Meier method. Subgroups (line of therapy, metastasis timing, hormone receptor (HR) status, treatment: olaparib, talazoparib, among others), germline *BRCA1*, *BRCA2* and *PALB2* mutations and adverse events (AEs) were analyzed. The median rwPFS was 6.2 months (95% CI, 4.8–7.9) and the median rwOS was 17.1 months (95% CI, 14.4–22.3). Line of therapy, HR status and treatment (olaparib versus talazoparib) appeared to especially affect both rwPFS and rwOS. Among patients with a reported germline mutation, 36.1% had a *BRCA1*, 62.9% a *BRCA2* and 1.0% a *PALB2* mutation. In summary, outcomes were comparable to those reported in pivotal trials despite later-line use of PARP-inhibitors in this analysis.

## Introduction

Poly (adenosine diphosphate-ribose) polymerase (PARP) inhibitors have been used in the treatment of patients with a human epidermal growth factor receptor type 2 (HER2)-negative advanced breast cancer (ABC) harboring a germline *BRCA1 or BRCA2* mutation since 2018^1,2^. The two genes are the most common homologous recombination deficiency (HRD) genes, responsible for the repair of deoxyribonucleic acid (DNA) double-strand breaks. In the OlympiAD trial, patients treated with olaparib had a median progression-free survival (PFS) of 7.0 (95% confidence interval [CI], 0.43–0.80) months and an overall survival (OS) of 19.3 months compared to 4.2 months (hazard ratio 0.58; 95% confidence interval [CI], 0.43–0.80) and 17.1 (hazard ratio 0.90, 95% CI 0.66–1.23) months in the control arm for patients receiving chemotherapy of physician’s choice (capecitabine, eribulin, vinorelbine)^2,3^. In EMBRACA, talazoparib improved median PFS from 5.6 to 8.6 months (HR, 0.54; 95% CI, 0.41–0.71) as compared with chemotherapy of physician’s choice (capecitabine, eribulin, gemcitabine, vinorelbine), while there was no difference in OS (19.3 versus 19.5 months; HR, 0.85; 95% CI, 0.670–1.073)^1,4^. In both trials treatment with a PARP-inhibitor was given as monotherapy without the addition of endocrine therapy in hormone-receptor (HR) positive disease.

The Phase 2 TBCRC 048 study reported response to therapy with olaparib for patients with a *PALB2* germline mutation (*N* = 24) with a median PFS of 9.6 months (90% CI, 8.3–12.4)^5,6^. The effect for patients with a somatic *BRCA-*mutation (*n* = 30) with a median PFS time of 5.6 months (90% CI, 3.0–8.3) was less pronounced^6^. The Talazoparib beyond BRCA trial showed activity of talazoparib in patients with a germline *PALB2* mutation. However, the sample size was limited (*N* = 5)^7^. Although approval was not amended for either drug for those indications, guidelines mention the possibility to treat patients with a germline *PALB2* mutation or a somatic *BRCA* mutation with olaparib^8–10^.

Frequent side effects of both olaparib and talazoparib are nausea, vomiting, diarrhea, fatigue, anemia, thrombocytopenia, neutropenia with its clinical manifestations, dyspnea, bleedings, and infections. About 0.73% (95% CI, 0.50–1.07) of patients develop a myelodysplastic syndrome due to PARP-inhibitor treatment^11^.

In the phase IIIb LUCY trial, which meant to include a population closer to routine clinical practice, olaparib demonstrated a median PFS time of 8.18 months in the germline BRCA-mutated cohort (95% CI, 6.97–9.17, *N* = 253)^12^. The LuciA-15 trial prospectively evaluated the use of PARP inhibitors in Argentina and México in 51 patients. Talazoparib was received by 62.7%, olaparib by 37.3% of patients^13^. PFS was 7.77 months (95% CI, 5.67–14.7) and OS was 26.97 months (95% CI, 13.50–NR). Few prospective real-world analyses with PARP inhibitors have been published and treatment reality in Germany regarding PARP-inhibitor therapy for ABC remains unknown. This analysis, therefore, aims to shed light on the treatment reality with PARP inhibitors in patients included into the prospective German PRAEGNANT registry, with a focus on subgroups, germline mutations and adverse events.

## Results

### Patient characteristics

Analyses were made within the final population of 152 patients receiving one of the two approved PARP-inhibitors in the advanced setting. Baseline patient and tumor characteristics are shown in Table 1. A total of 90.8% (*N* = 138) of patients received olaparib and 9.2% (*N* = 14) received talazoparib. 35.5% (*N* = 54) of patients had triple-negative disease and 64.5% (*N* = 98) of patients had HR-positive, HER2-negative disease. The mean age was 51.0 (standard deviation [SD], 11.2) years, and the mean BMI was 24.4 (SD 5.1) kg/m^2^. The majority of patients had an ECOG status of 0 or 1, with 55.5% (*N* = 76) and 36.5% (*N* = 50), respectively. In total, 12.5% (*N* = 19) received PARP inhibitors in the first line of therapy for ABC, 34.9% (*N* = 53) in the second line, and 19.1% (*N* = 29) in the third line, with fewer patients receiving the PARP-inhibitor in later therapy lines. Visceral metastasis was observed in 66.9% (*N* = 99) of patients; 16.2% (*N* = 24) had brain metastasis and 4.7% (*N* = 7) bone-only disease. A de novo metastatic disease was identified in 25.2% (*N* = 37) of patients, 41.5% (*N* = 61) developed metastatic disease within 60 months, and 33.3% (*N* = 49) more than 60 months after primary diagnosis of breast cancer. Concomitant diseases at the start of PARP-inhibitor treatment were present in 65.6% (*N* = 99), with 27.7% (*N* = 42) of the patients having three or more. The median observation time for rwPFS was 6.1 months (interquartile range (IQR), 3.0–10.2 months) and 12.8 (IQR, 7.6–23.9) months for rwOS.

**Table 1:** Patient and tumor characteristics, showing mean and standard deviation (SD), median and interquartile range (IQR), or frequency and percentage

		All patients (*N* = 152)
Age (years)	Mean (SD)	51.0 (11.2)
	Median (IQR)	51.0 (43.0, 58.0)
	Up to 49	68 (44.7)
	50–64	68 (44.7)
	65+	16 (10.5)
	Missing	0
BMI (kg/m^2^)	Mean (SD)	24.4 (5.1)
	Median (IQR)	23.5 (20.5, 27.5)
	<18.5 (Underweight)	11 (8.3)
	18.5–24.9 (Normal)	70 (52.6)
	25.0–<30 (Overweight)	34 (25.6)
	>30 (Obese)	18 (13.5)
	Missing	19
ECOG performance status	0	76 (55.5)
	1	50 (36.5)
	2	9 (6.6)
	3	2 (1.5)
	Missing	15
Grading	G1	4 (2.9)
	G2	63 (45.7)
	G3	71 (51.4)
	Missing	14
Line of therapy	1	19 (12.5)
	2	53 (34.9)
	3	29 (19.1)
	4	18 (11.8)
	5	15 (9.9)
	6	7 (4.6)
	7	5 (3.3)
	8	6 (3.9)
	Missing	0
Metastasis pattern	Brain	24 (16.2)
	Visceral	99 (66.9)
	Bone only	7 (4.7)
	Others	18 (12.2)
	Missing	4
Metastasis status	De novo metastasis	37 (25.2)
	Metastasis ≤60 months	61 (41.5)
	Metastasis >60 month	49 (33.3)
	Missing	5
HR-status	Positive	98 (64.5)
	Negative	54 (35.5)
	Missing	0
Treatment	Olaparib	138 (90.8)
	Talazoparib	14 (9.2)
	Missing	0
Concomitant diseases	0	52 (34.4)
	1	24 (15.9)
	2	33 (21.9)
	3–5	34 (22.5)
	>5	8 (5.3)
	Missing	1

### Real-world progression-free survival

**Fig. 1:**
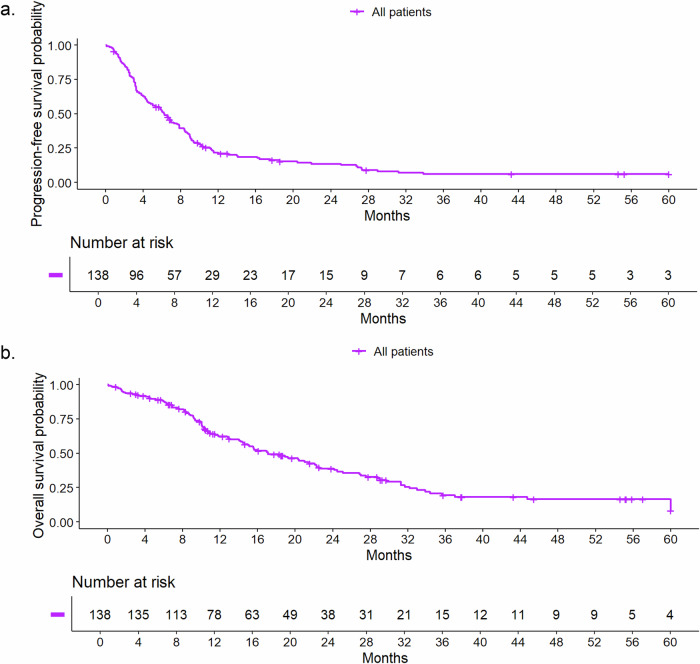
Real-world progression-free and overall survival for all patients^1^. **a** Real-world progression-free survival for all patients^1^ (**b**) Real-world overall survival for all patients^1^. ^1^The number of patients at the start of the observation in the Kaplan-Meier curves (*n* = 138) was lower than the actual patients included (*n* = 152) due to left truncation.

**Fig. 2:**
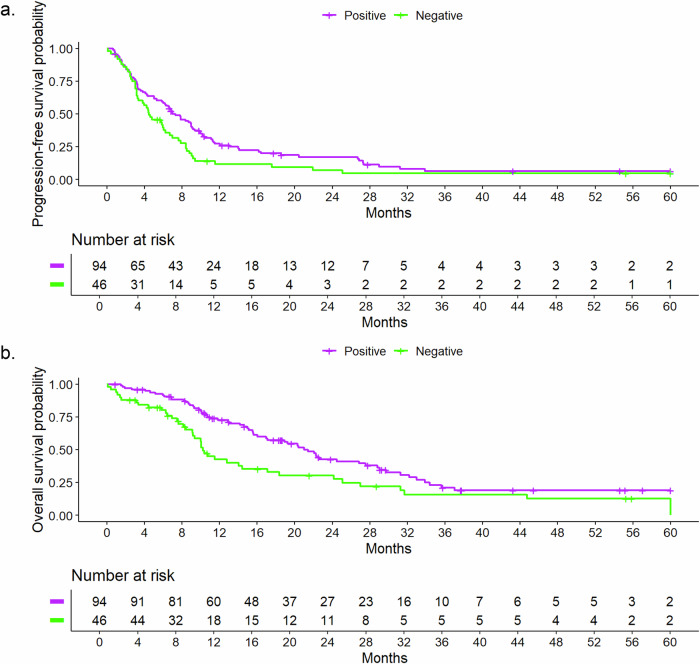
Real-world progression-free and overall survival relative to hormone receptor (HR) status. **a** Real-world progression-free survival relative to hormone receptor (HR) status. **b** Real-world overall survival relative to hormone receptor (HR) status.

**Fig. 3:**
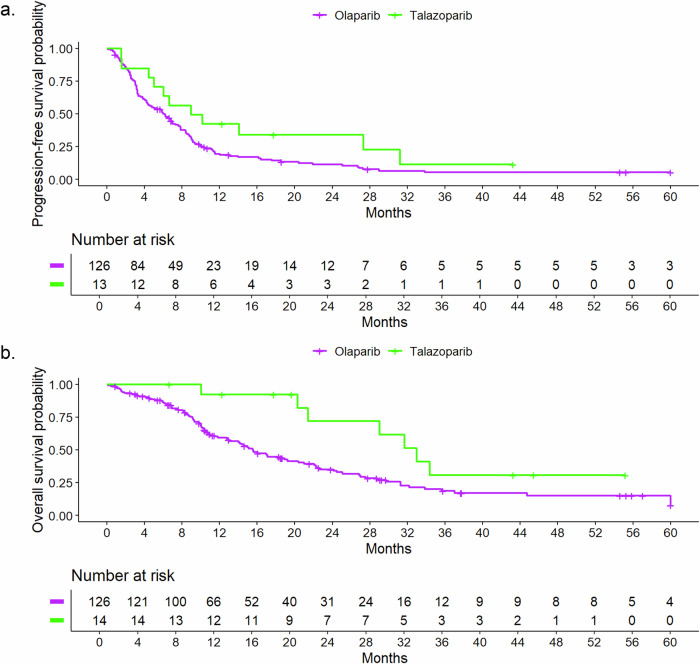
Real-world progression-free survival and overall survival relative to treatment. **a** Real-world progression-free survival relative to treatment. **b** Real-world overall survival relative to treatment.

The median rwPFS time was 6.2 months (95% CI, 4.8–7.9), the Kaplan–Meier graph is shown in Fig. 1a. Six-month, 12-month, 24-month, and 60-month rwPFS rates with 95% CI with regard to different cofactors are presented in Table 2. A trend was seen towards a better prognosis for patients receiving the PARP-inhibitor in the second line of therapy with a median rwPFS time of 9.0 months (95% CI, 7.3–11.3) compared to 7.9 months (95% CI, 4.4–21.9) in the first line, 6.3 months (95% CI, 4.1–8.8) in the third line and 3.2 months (95% CI, 2.5–4.4) in the fourth and later lines and for HR-positive disease with a median rwPFS time of 7.3 months (95% CI, 6.0–9.2) compared to 4.5 months (95% CI, 3.4–6.7) for triple-negative disease. The Kaplan-Meier curve for rwPFS according to HR status is shown in Fig. 2a. For HR-positive disease, a longer rwPFS in earlier therapy lines was observed, with rwPFS decreasing with each additional line of therapy. The longest rwPFS occurred in the first line therapy with 18.5 months (95% CI, 7.9–NA; *N* = 7) (Fig. S3a). For triple-negative disease rwPFS in the first and second line was similar within this subgroup, with poorer rwPFS from the third line onwards (Table S1 and Fig. S3b). Furthermore, bone-only disease had the longest median rwPFS time compared to all other metastasis sites (Fig. S1f). The median rwPFS time for patients treated with olaparib was 6.0 months (95% CI, 4.4–7.6) compared to 9.0 months (95% CI, 6.0–NA) for patients treated with talazoparib (Fig. 3a). However, only 14 patients in our cohort were treated with talazoparib, compared to 138 patients treated with olaparib. All remaining Kaplan-Meier curves for rwPFS based on different cofactors are shown in the supplementary information (Fig. S1a–h).

**Table 2:** Median real-world progression-free survival times and survival rates

Characteristic		*N*	Events	Median survival time in months (95% CI)	Survival rate (95% CI)			
					6-month	12-month	24-month	60-month
All patients		152	134	6.2 (4.8, 7.9)	0.52 (0.45, 0.61)	0.22 (0.16, 0.30)	0.14 (0.09, 0.21)	0.06 (0.03, 0.12)
Age	Up to 49	68	60	5.9 (3.3, 7.0)	0.48 (0.38, 0.62)	0.19 (0.12, 0.32)	0.12 (0.06, 0.24)	0.06 (0.02, 0.17)
	50 - 64	68	60	6.8 (4.4, 9.2)	0.53 (0.42, 0.66)	0.20 (0.12, 0.32)	0.12 (0.06, 0.24)	0.06 (0.02, 0.17)
	65+	16	13	8.8 (4.2, NA)	0.69 (0.49, 0.96)	0.41 (0.23, 0.75)	0.25 (0.10, 0.63)	0.00^b^
BMI	Underweight	11	11	6.2 (3.8, NA)	0.55 (0.32, 0.94)	0.18 (0.05, 0.64)	0.09 (0.01, 0.59)	0.00^b^
	Normal	70	59	5.9 (4.1, 8.5)	0.49 (0.39, 0.62)	0.28 (0.19, 0.41)	0.12 (0.05, 0.24)	0.06 (0.02, 0.20)
	Overweight	34	30	6.7 (4.5, 10.2)	0.59 (0.44, 0.78)	0.18 (0.09, 0.36)	0.18 (0.09, 0.36)	0.11 (0.04, 0.29)
	Obese	18	16	6.3 (3.3, 10.4)	0.56 (0.37, 0.84)	0.07 (0.01, 0.45)	0.00^a^	0.00^b^
ECOG performance status	0	76	66	7.0 (6.0, 9.0)	0.60 (0.50, 0.72)	0.27 (0.18, 0.39)	0.18 (0.11, 0.29)	0.08 (0.03, 0.19)
	>0	61	54	5.0 (3.2, 7.9)	0.45 (0.34, 0.60)	0.13 (0.07, 0.27)	0.03 (0.00, 0.18)	0.03 (0.00, 0.18)
Grading	G1 or G2	67	59	7.0 (5.3, 9.1)	0.56 (0.45, 0.70)	0.21 (0.13, 0.34)	0.13 (0.07, 0.26)	0.06 (0.02, 0.19)
	G3	71	62	4.8 (3.4, 7.9)	0.45 (0.35, 0.58)	0.22 (0.14, 0.35)	0.14 (0.08, 0.26)	0.07 (0.03, 0.18)
Line of therapy	1	19	17	7.9 (4.4, 21.9)	0.57 (0.39, 0.85)	0.42 (0.24, 0.71)	0.14 (0.04, 0.48)	0.00^b^
	2	53	39	9.0 (7.3, 11.3)	0.69 (0.57, 0.83)	0.31 (0.20, 0.47)	0.23 (0.14, 0.40)	0.17 (0.08, 0.34)
	3	29	27	6.3 (4.1, 8.8)	0.55 (0.40, 0.76)	0.18 (0.08, 0.41)	0.09 (0.02, 0.32)	0.00^b^
	4+	51	50	3.2 (2.5, 4.4)	0.33 (0.22, 0.49)	0.08 (0.03, 0.20)	0.06 (0.02, 0.17)	0.02 (0.00, 0.14)
Metastasis pattern	Brain	24	20	5.8 (3.3, 11.5)	0.46 (0.30, 0.72)	0.23 (0.11, 0.50)	0.17 (0.07, 0.45)	0.06 (0.01, 0.37)
	Visceral	99	91	6.0 (4.2, 8.4)	0.50 (0.41, 0.61)	0.17 (0.11, 0.27)	0.10 (0.06, 0.19)	0.05 (0.02, 0.13)
	Bone only	7	4	17.6 (6.8, NA)	0.86 (0.63, 1.00)	0.51 (0.24, 1.00)	0.26 (0.05, 1.00)	0.26 (0.05, 1.00)
	Others	18	15	6.2 (3.8, 25.1)	0.60 (0.41, 0.88)	0.30 (0.14, 0.62)	0.15 (0.04, 0.51)	0.07 (0.01, 0.47)
Metastasis status	De novo metastasis	37	32	6.7 (4.5, 9.2)	0.57 (0.43, 0.75)	0.19 (0.10, 0.38)	0.12 (0.05, 0.31)	0.08 (0.02, 0.28)
	Metastasis ≤ 60 months	61	54	5.9 (3.4, 7.9)	0.49 (0.38, 0.64)	0.20 (0.12, 0.34)	0.12 (0.06, 0.25)	0.05 (0.01, 0.17)
	Metastasis >60 month	49	43	7.0 (4.4, 11.1)	0.55 (0.42, 0.71)	0.27 (0.17, 0.43)	0.17 (0.09, 0.33)	0.06 (0.02, 0.22)
Concomitant diseases	0 or 1	76	70	6.0 (3.6, 7.9)	0.51 (0.41, 0.64)	0.19 (0.12, 0.31)	0.08 (0.04, 0.19)	0.03 (0.00, 0.15)
	>1	76	63	6.8 (5.0, 9.0)	0.53 (0.43, 0.66)	0.24 (0.16, 0.37)	0.19 (0.12, 0.31)	0.10 (0.04, 0.21)
HR-Status	HR+	98	84	7.3 (6.0, 9.2)	0.59 (0.50, 0.70)	0.27 (0.20, 0.38)	0.17 (0.11, 0.27)	0.07 (0.03, 0.16)
	HR−	54	49	4.5 (3.4, 6.7)	0.40 (0.28, 0.55)	0.12 (0.05, 0.25)	0.07 (0.02, 0.20)	0.05 (0.01, 0.17)
Treatment	Olaparib	138	122	6.0 (4.4, 7.6)	0.51 (0.43, 0.60)	0.19 (0.14, 0.28)	0.11 (0.07, 0.19)	0.05 (0.02, 0.12)
	Talazoparib	14	11	9.0 (6.0, NA)	0.71 (0.50, 0.99)	0.42 (0.23, 0.78)	0.34 (0.16, 0.72)	0.11 (0.02, 0.66)

*CI* confidence interval, *BMI* body mass index, *ECOG* Eastern Cooperative Oncology Group, *HR* hormone receptor, NA not available.

^a^No patient reached an observation time of 24 months.

^b^No patient reached an observation time of 60 months.

### Real-world overall survival

The median rwOS time was 17.1 months (95% CI, 14.4–22.3). Figure 1b presents the Kaplan–Meier survival graphs for rwOS. Six-month, 12-month, 24-month, and 60-month rwOS rates with 95% CI with regard to different cofactors are presented in Table 3. Under the limitation of small subgroups, first-line therapy and bone-only disease were linked to longer rwOS compared to PARP-inhibitor therapy in later therapy lines and visceral or brain metastases. The median rwOS for patients with HR-positive disease was 21.4 months (95% CI, 17.0-28.8) compared to 10.2 months (95% CI, 9.2–18.4) for patients with triple-negative disease (Fig. 2b). The median rwOS time for patients treated with olaparib (*N* = 138) was 15.6 months (95% CI, 12.9–21) compared to 33.0 months (95% CI, 21.4–NA) for patients treated with talazoparib (*N* = 14, Fig. 3b). Similar to rwPFS, for HR-positive disease a trend towards longer rwOS in earlier therapy lines was observed (Fig. S4a). Patients with triple-negative disease treated with a PARP-inhibitor in the first and second line had similar rwOS. From the third line onward, rwOS declined (Fig. S4b and Table S2). Further rwOS Kaplan–Meier graphs based on different cofactors are presented in the supplementary information (Fig. S2a–h).

**Table 3:** Median real-world overall survival times and survival rates

Characteristic		*N*	Events	Median survival time in months (95% CI)	Survival rate (95% CI)			
					6-month	12-month	24-month	60-month
All patients		152	100	17.1 (14.4, 22.3)	0.88 (0.82, 0.93)	0.62 (0.55, 0.71)	0.39 (0.31, 0.49)	0.08 (0.03, 0.24)
Age	Up to 49	68	52	15.6 (10.1, 20.4)	0.80 (0.71, 0.90)	0.50 (0.39, 0.64)	0.30 (0.20, 0.45)	0.06 (0.01, 0.30)
	50–64	68	40	22.1 (14.4, 31.2)	0.92 (0.86, 0.99)	0.73 (0.63, 0.85)	0.46 (0.34, 0.62)	0.09 (0.02, 0.43)
	65+	16	8	19.3 (14.6, NA)	1.00 (1.00, 1.00)	0.71 (0.51, 0.99)	0.48 (0.27, 0.84)	0.32 (0.12, 0.85)
BMI	Underweight	11	7	15.0 (9.1, NA)	0.90 (0.73, 1.00)	0.60 (0.36, 1.00)	0.30 (0.10, 0.90)	0.15 (0.03, 0.88)
	Normal	70	43	20.4 (13.0, 31.2)	0.85 (0.77, 0.94)	0.62 (0.51, 0.76)	0.42 (0.30, 0.57)	0.11 (0.04, 0.34)
	Overweight	34	23	15.1 (10.5, NA)	0.88 (0.78, 1.00)	0.62 (0.47, 0.82)	0.40 (0.26, 0.63)	0.09 (0.02, 0.50)
	Obese	18	13	21.0 (9.3, NA)	0.83 (0.68, 1.00)	0.58 (0.38, 0.88)	0.29 (0.13, 0.65)	0.10 (0.02, 0.58)
ECOG performance status	0	76	48	22.2 (17.0, 29.0)	0.93 (0.88, 0.99)	0.66 (0.55, 0.78)	0.47 (0.36, 0.62)	0.00^1^
	>0	61	42	14.1 (9.8, 19.3)	0.78 (0.68, 0.89)	0.55 (0.44, 0.70)	0.23 (0.14, 0.40)	0.13 (0.05, 0.31)
Grading	G1 or G2	67	46	17.1 (14.4, 24.5)	0.89 (0.82, 0.97)	0.67 (0.57, 0.80)	0.35 (0.24, 0.51)	0.12 (0.05, 0.28)
	G3	71	43	15.6 (10.3, 33.9)	0.85 (0.77, 0.94)	0.55 (0.44, 0.69)	0.44 (0.33, 0.59)	0.09 (0.02, 0.46)
Line of therapy	1	19	8	44.7 (18.4, NA)	0.89 (0.75, 1.00)	0.71 (0.52, 0.96)	0.63 (0.43, 0.92)	0.37 (0.14, 0.92)
	2	53	30	24.2 (18.9, 33.0)	1.00 (1.00, 1.00)	0.79 (0.68, 0.91)	0.51 (0.37, 0.70)	0.07 (0.01, 0.39)
	3	29	22	12.8 (10.1, 19.3)	0.93 (0.84, 1.00)	0.50 (0.34, 0.74)	0.17 (0.07, 0.41)	0.13 (0.04, 0.36)
	4+	51	40	14.2 (9.1, 22.3)	0.71 (0.60, 0.85)	0.50 (0.38, 0.67)	0.32 (0.20, 0.49)	0.09 (0.03, 0.25)
Metastasis pattern	Brain	24	15	15.0 (11.5, NA)	0.77 (0.61, 0.97)	0.62 (0.45, 0.87)	0.35 (0.18, 0.65)	0.00^1^
	Visceral	99	73	15.6 (12.8, 22.1)	0.88 (0.81, 0.94)	0.60 (0.51, 0.71)	0.34 (0.25, 0.46)	0.09 (0.03, 0.25)
	Bone only	7	2	44.7 (NA, NA)	1.00 (1.00, 1.00)	0.83 (0.58, 1.00)	0.83 (0.58, 1.00)	0.00^1^
	Others	18	9	25.1 (10.5, NA)	0.94 (0.84, 1.00)	0.65 (0.44, 0.96)	0.55 (0.34, 0.91)	0.22 (0.07, 0.72)
Metastasis status	De novo metastasis	37	24	20.4 (14.1, 31.2)	0.92 (0.83, 1.00)	0.65 (0.51, 0.83)	0.43 (0.28, 0.65)	0.00^1^
	Metastasis ≤60 months	61	44	15.6 (10.5, 22.1)	0.84 (0.75, 0.94)	0.55 (0.43, 0.70)	0.31 (0.21, 0.48)	0.00^a^
	Metastasis >60 month	49	28	22.6 (15.6, 37.0)	0.87 (0.78, 0.97)	0.74 (0.63, 0.88)	0.48 (0.35, 0.67)	0.26 (0.14, 0.48)
Concomitant diseases	0 or 1	76	55	15.1 (11.5, 20.3)	0.82 (0.74, 0.91)	0.59 (0.48, 0.71)	0.31 (0.22, 0.45)	0.07 (0.02, 0.34)
	>1	76	45	22.3 (15.6, 29.7)	0.93 (0.88, 0.99)	0.66 (0.56, 0.79)	0.47 (0.36, 0.62)	0.09 (0.02, 0.43)
HR-Status	HR+	98	60	21.4 (17.0, 28.8)	0.92 (0.86, 0.97)	0.73 (0.64, 0.82)	0.43 (0.33, 0.55)	0.19 (0.11, 0.32)
	HR−	54	40	10.2 (9.2, 18.4)	0.80 (0.70, 0.92)	0.42 (0.30, 0.60)	0.30 (0.19, 0.48)	0.00^1^
Treatment	Olaparib	138	93	15.6 (12.9, 21)	0.86 (0.81, 0.92)	0.59 (0.51, 0.69)	0.35 (0.27, 0.45)	0.08 (0.02, 0.23)
	Talazoparib	14	7	33.0 (21.4, NA)	1.00 (1.00, 1.00)	0.92 (0.79, 1.00)	0.72 (0.49, 1.00)	0.31 (0.12, 0.79)

*CI* confidence interval, *BMI* body mass index, *ECOG* Eastern Cooperative Oncology Group, *HR* hormone receptor, NA not available.

^a^No patient reached an observation time of 60 months.

### Mutations

The mutation status with corresponding frequencies and percentages for *BRCA1*, *BRCA2*, and *PALB2* are presented in Table S3. Among patients with a germline mutation, 36.1% (*N* = 35) had a *BRCA1*, 62.9% (*N* = 61) a *BRCA2* and 1.0% (*N* = 1) a *PALB2* mutation (Table S3). The exact Human Genome Variation Society nomenclature term for every patient is shown in Tables S4 and S5 regarding germline *BRCA1* and *BRCA2* mutations. The corresponding clinical variation classifications are listed in Tables S6 andS7.

### Serious adverse and adverse events

In the summary of all adverse events (AEs) by the Medical Dictionary for Regulatory Activities Preferred Terms (MedDRA PT), fatigue was the most frequently reported AEs, occurring in 8.6% (*N* = 13) of patients, followed by nausea (*N* = 11 events) and pain in extremity (*N* = 11 events) in 7.2% (*N* = 11) and 5.3% (*N* = 8) of patients, respectively (Table S8). Grades 3–5 AEs and serious AEs are also summarized separately in the supplementary information (Tables S9 and S10).

## Discussion

Our real-world analysis of PARP-inhibitors in patients with HER2-negative ABC shows a median rwPFS time of 6.2 months (95% CI, 4.8–7.9) and a median rwOS time of 17.1 months (95% CI, 14.4–22.3). Line of therapy, HR status, and treatment (Olaparib versus talazoparib) appeared to especially affect both rwrPFS and rwOS.

This efficacy seemed to be comparable to the efficacy in the large randomized phase III trials with a slightly shorter median rwPFS. Compared to the OlympiAD trial however, patients in our real-world cohort were treated in later therapy lines. As prognosis worsens with therapies given in later therapy lines, this might be an importing contributing factor^14^. Also, over time and after approval of the PARP-inhibitors, the treatment landscapes both in HR-positive and triple-negative ABC changed, possibly increasing the number of more heavily pre-treated patients starting with the PARP-inhibitor therapy. HR-positive breast cancer patients in OlympiAD did not receive a CDK4/6i prior to olaparib. However, CDK4/6i therapy is the standard-of-care first-line therapy for HR-positive ABC today. The same stands true for triple-negative diseases, where chemotherapy gets replaced by antibody drug conjugates (ADCs), which get approved in ever earlier therapy lines and are on the cusp of being used for first-line treatment. Although OlympiAD and EMBRACA did not show a statistically significant OS benefit, patients in the first line had a longer median OS with olaparib compared to therapy of physician’s choice (22.6 versus 14.7 months; HR 0.55, 95% CI 0.33–0.95)^15^. Patients treated with talazoparib in the EMBRACA trial resembled a more treatment-naïve patient cohort^1,4^. While the median line of therapy with PARP inhibitors in OlympiAD and EMBRACA was the second line, patients in our cohort received the PARP inhibitor, on average, in the third line of therapy. All the more, our data underscores the effectiveness of the two medications, showing similar rwPFS and rwOS compared with the pivotal trials despite being administered, on average, in a later line of therapy.

54.4% (*N* = 137) of patients in the LUCY trial received olaparib in the first line and 52% (*N* = 131) were HR-positive^16^. In comparison, in our cohort only 12.5% (*N* = 19) received the PARP inhibitor in the first line and 64.5% (*N* = 98) were HR-positive, which makes comparisons difficult. Compared to the LuciA-15 trial, with a limited sample size of only 51 patients, our cohort shows slightly shorter median rwPFS and rwOS. To a larger extent, patients included in the LuciA-15 trial received talazoparib (62.7%) compared to olaparib (37.3%)^13^. However, it remains unclear whether the longer observed median rwPFS and rwOS in this small cohort result from the therapy received.

The median rwOS in the first-line was 44 months in our cohort, warranting further exploration of the timepoint of usage in the therapy landscape, given that for a treatment with CDK4/6 inhibitors in patients with a *BRCA2* mutation, some kind of resistance has been described with shorter PFS times than in patients with a wildtype genotype^17^. The adverse events described in our cohort are similar to those reported in the pivotal trials, but were reported at a much lower frequency. Ascertainment bias likely resulted in and under-reporting in our real-world trial similar to other real-world studies^18^.

Mutations in HER2-negative ABC have been described before in the PRAEGNANT registry^19^. *BRCA1/BRCA2* mutations were found in 5% of patients. Other HRD mutations were seen in 2.9% of patients; 5.8% of the patients had mutations in other DNA repair genes, and 1.6% of patients in other cancer risk genes.^5,6^. With only one reported *PALB2* mutation carrier in our cohort, germline *BRCA1/BRCA2* mutations remain the main indication for PARP-inhibitor therapy. Missing mutation information data must be noted as a limitation, however.

In our study, 17 patients received their PARP inhibitor in combination with endocrine therapy. Although endocrine therapy for HR-positive HER2-negative patients was not part of the treatment in OlympiAD and EMBRACA, the phase 2 DOLAF trial evaluated the combination of olaparib, fulvestrant, plus durvalumab, 67 of whom had a reported germline *BRCA1/BRCA2* mutation^20^. The PFS in this group was 12.6 months (95% CI, 8.2–16.7). Furthermore, in the early setting, in the OlympiA trial, which demonstrated an OS-benefit, 86.9% of HR-positive patients received endocrine therapy in combination with Olaparib^21,22^. The combination of PARP-inhibitors in combination with endocrine based therapies is under investigation in more recent trials like the Evopar-BR01 study (NCT06380751)^23^.

Our study has several limitations. One is the still relatively small sample size, especially within subgroups. Furthermore, as is common with prospective registry trials, AE reporting has to be interpreted with caution due to probable underreporting, permitting only signal detection rather than a true safety assessment^24^. The most frequently reported adverse events in registrational trials (e.g., anemia, nausea, and fatigue) were nonetheless documented in the registry. A reasonable benefit-risk assessment of PARP inhibitors, taking into account not only survival outcomes, but also toxicity and quality-of-life during therapy, is therefore not possible with this study. With respect to mutation status, somatic mutations were not evaluated and for 36.2% of patients, mutation status remained unclear in the present real-world analysis. Even though guidelines mention the possibility to treat patients with germline *PALB2* mutations, with only one patient included, statements in our analysis can be made for patients with *BRCA* mutations only. Our data confirms the results from the registrational trials of olaparib and talazoparib, showing very similar median rwPFS and rwOS times. With good tolerability, the RWD supports the recommendation for germline *BRCA1* and *BRCA2* testing in order to enable patients to receive therapy with a PARP inhibitor. Further research is needed to explore resistance mechanisms emerging during PARP-inhibitor use and to address questions of PARP-inhibitor sequencing and PARP-inhibitor after PARP-inhibitor use in ABC.

## Methods

### The PRAEGNANT research network

The PRAEGNANT (Prospective Academic Translational Research Network for the Optimization of the Oncological Health Care Quality in the Adjuvant and Advanced/Metastatic Setting, NCT02338167^25^,) study is an ongoing, prospective breast cancer registry. Documentation is similar to that of a clinical trial. The first patient was recruited in July 2014. The PRAEGNANT registry aims to assess treatment patterns, to investigate quality of life and survivorship, to answer translational research questions, and finally, to identify patients’ eligibility for clinical trials or specific targeted treatments^25–28^. Patients can be included at any given point during the course of their disease. Follow-up assessments for the advanced setting are updated every three months until month 24 and thereafter every 6 months in case there is no progression or change of therapy within three months of observation. Furthermore, biomaterials from blood and tumor biopsies are collected for research purposes^25^. The study was conducted according to the guidelines of the Declaration of Helsinki, and was approved by the respective German ethics committees (ethical approval number: 234/2014BO1, first approval on 17 June 2014, approval of Amendment 1 on 11 June 2015, approval of Amendment 2 on 18 March 2019; approval of Amendment 3 on 12 October 2022; approval of Amendment 4 on 14 April 2025; Ethics Committee of the Medical Faculty, University of Tübingen, Tübingen, Germany). All patients included in the present study provided informed consent.

### Patients

**Fig. 4:**
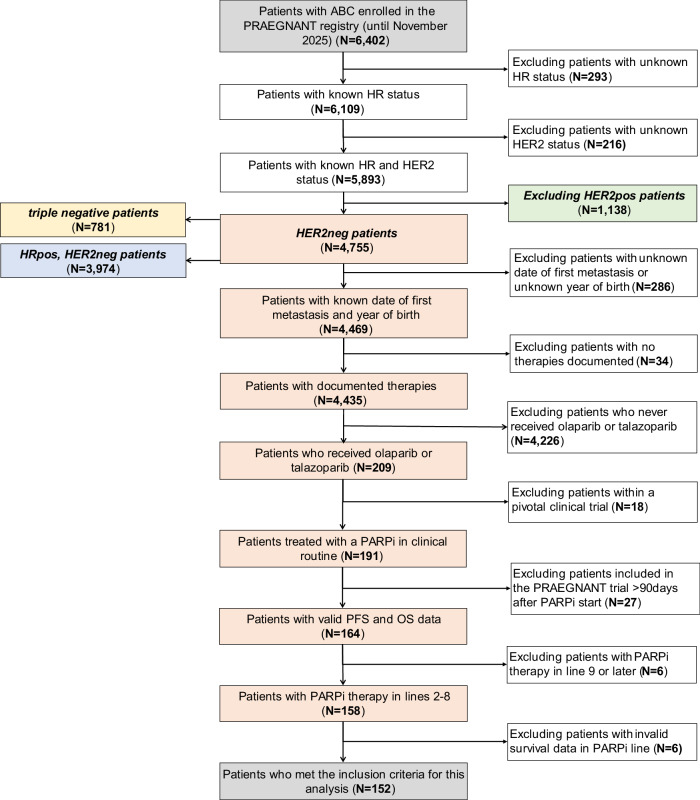
Patient inclusion and exclusion flow chart.

At the time of data cut-off (October 07, 2025), 6402 ABC patients were registered in the PRAEGNANT registry. Of these, 5893 patients had both documented HR and HER2 status. Of this overall population, 1138 patients were excluded due to HER2-positive disease, leaving 4755 patients, of whom 781 patients had triple-negative disease and 3974 patients had HR-positive, HER2-negative disease. A total of 286 patients had to be excluded due to missing documentation of the date of first metastasis or unknown year of birth, 34 patients had no documented therapy, leaving 4435 patients with documented therapies. After excluding 4226 patients who did not receive a PARP inhibitor, 209 patients remained in the dataset. Of those, 18 patients had to be excluded due to PARP-inhibitor therapy in a pivotal study. Likewise, 27 patients were included in the PRAEGNANT registry after the start of PARP-inhibitor therapy and had to be excluded. In order to maintain a more homogeneous study population six patients receiving PARP-inhibitors in therapy line 9 or afterwards were excluded. Six further patients had invalid PFS or OS follow-up data and were thus excluded. Consequently, 152 patients were included in the final patient population. The patient flow chart is shown in Fig. 4.

### Data ascertainment

Data was collected by trained personnel and documented in an electronic case report form^25^. Automated plausibility checks issued and addressed queries regarding the research question, and on-site monitoring was performed. Data not recorded in everyday clinical work were prospectively collected using structured questionnaires completed on paper (epidemiological data such as family history, cancer risk factors, quality of life, nutrition and lifestyle items, and psychological health).

### Definition of grading, HR, and HER2 status

The definition of HR status, HER2 status, and grading has been described previously^26^. In summary, if a biomarker assessment of the metastatic site was available, this receptor status was used for the analysis. If there was no information for metastases, the latest biomarker results from the primary tumor were used. Additionally, all patients who received endocrine therapy in the metastatic setting were assumed to be HR-positive, and all patients who had ever received anti-HER2 therapy were considered to be HER2-positive. There was no central review of biomarkers. The study protocol recommended assessing estrogen-receptor and progesterone-receptor status as positive if ≥1% of cancer cells were stained. A positive HER2 status was defined by an immunohistochemistry score of 3+ or positive fluorescence in situ hybridization/chromogenic in situ hybridization.

### Assessment of germline mutations

Germline mutations were specifically queried in the current analysis to improve documentation quality. Somatic mutations were not evaluated in the present analysis.

### Statistical analysis

Patient and tumor characteristics were described using summary statistics. Mean and standard deviation and median and interquartile range (IQR) are calculated for continuous variables, whereas frequencies and percentages are used for categorical variables.

Real-world PFS (rwPFS) was defined as the time from the start of therapy to the earliest occurrence of disease progression (distant-metastasis, local recurrence, or death from any cause), or the last known date the patient was progression-free. The analysis was left-truncated at the time of study entry if entry occurred after the initiation of therapy. Patients without an event were censored at the last follow-up or at 60 months (five years), whichever came first. Real-world OS (rwOS) was defined in a similar manner.

The primary objective was to investigate rwPFS in patients receiving olaparib or talazoparib. Survival rates with 95% CIs and median survival times were estimated using the Kaplan-Meier product-limit method. Subgroup analyses were conducted based on the following variables: age (categorical; up to and including 49 years, 50 to 64 years, 65+ years), body mass index (BMI, categorical; underweight, <18.5 (kg/m²); normal, 18.5–24.9 (kg/m²); overweight, 25–29.9; obese, >29.9 (kg/m²)), ECOG performance status (categorical; 0, >0), grading (ordinal; G1/G2, G3), line of therapy (ordinal; 1, 2, 3, 4 or more), metastasis pattern (categorical; brain, visceral, bone only, others), metastasis status (categorical; de novo metastasis, metastasis ≤ 60 months after primary diagnosis, metastasis >60 months after primary diagnosis), concomitant diseases (categorical; 0 or 1, >1), hormone receptor status (HR, categorical; positive, negative) and treatment (categorical; olaparib, talazoparib). The variables used for the subgroup analyses were defined at the time of initiation of PARP-inhibitor therapy or, where applicable, at the most recent documented assessment before PARP-inhibitor use for age, BMI, ECOG performance status, grading, metastatic pattern, concomitant diseases and hormone receptor (HR) status. For HR status, if none was available at or before PARP-inhibitor initiation, it was further analyzed if any status was documented in lines 1–4. The 95% CI for the median survival time was calculated using the Brookmeyer and Crowley method^29^. The same approach was applied for rwOS. Cox proportional hazards regression analyses or other statistical testing were not performed due to the limited sample size.

Calculations were carried out using the R system for statistical computing (version 4.3.0; R Development Core Team, Vienna, Austria, 2023).

## Supplementary information

Supplementary information

## Data Availability

The datasets used and analyzed during the current study are available upon reasonable request in the context of a research project. Proposals are evaluated by the PRAEGNANT scientific board. In case of approval, data is available from the corresponding author.
