# Supplementary material for: PARP inhibition with olaparib and talazoparib for HER2-negative advanced breast cancer—Results from the prospective PRAEGNANT registry
Source: NPJ Breast Cancer. 2026 Apr 11;12:60. doi: 10.1038/s41523-026-00947-8 (PMC13087296; doi:10.1038/s41523-026-00947-8)

# Supplementary information

**Table S1**: Real-world median progression-free survival times and survival rates relative to hormone receptor (HR) status and line of therapy.

| **HR status** | **Line of therapy** | **N** | **Events** | **Median survival time in months  (95%-CI)** | **Survival rate (95%-CI)** | | | |
| --- | --- | --- | --- | --- | --- | --- | --- | --- |
|  |  |  |  |  | **6-month** | **12-month** | **24-month** | **60-month** |
| HR+ | 1 | 7 | 6 | 18.5 (7.9, NA) | 0.86 (0.63, 1.00) | 0.71 (0.45, 1.00) | 0.36 (0.12, 1.00) | 0.00^3^ |
|  | 2 | 35 | 24 | 9.9 (7.3, 20.5) | 0.70 (0.56, 0.87) | 0.39 (0.25, 0.60) | 0.27 (0.15, 0.50) | 0.22 (0.10, 0.46) |
|  | 3 | 18 | 16 | 7.9 (6.3, 26.7) | 0.78 (0.61, 1.00) | 0.29 (0.14, 0.63) | 0.15 (0.04, 0.51) | 0.00^3^ |
|  | 4+ | 38 | 38 | 3.2 (2.5, 6.8) | 0.36 (0.24, 0.55) | 0.08 (0.03, 0.23) | 0.05 (0.01, 0.02) | 0.00^3^ |
| HR− | 1 | 12 | 11 | 5.9 (3.4, NA) | 0.41 (0.21, 0.81) | 0.25 (0.09, 0.66) | 0.00^2^ | 0.00^3^ |
|  | 2 | 18 | 15 | 7.6 (5.8, 11.5) | 0.66 (0.47, 0.92) | 0.13 (0.04, 0.48) | 0.13 (0.04, 0.48) | 0.07 (0.01, 0.43) |
|  | 3 | 11 | 11 | 3.1 (2.1, NA) | 0.18 (0.05, 0.63) | 0.00^1^ | 0.00^2^ | 0.00^3^ |
|  | 4+ | 13 | 12 | 3.2 (1.8, NA) | 0.23 (0.09, 0.62) | 0.08 (0.01, 0.51) | 0.08 (0.01, 0.51) | 0.08 (0.01, 0.51) |

*CI* confidence interval, *HR* hormone receptor, NA not available

^1^No patient reached an observation time of 12 months

^2^No patient reached an observation time of 24 months

^3^No patient reached an observation time of 60 months

**Table S2**: Median real-world overall survival times and survival rates relative to hormone receptor (HR) status and line of therapy.

| **HR status** | **Line of therapy** | **N** | **Events** | **Median survival time in months  (95%-CI)** | **Survival rate (95%-CI)** | | | |
| --- | --- | --- | --- | --- | --- | --- | --- | --- |
|  |  |  |  |  | **6-month** | **12-month** | **24-month** | **60-month** |
| HR+ | 1 | 7 | 1 | -^1^ | 1.00 (1.00, 1.00) | 0.83 (0.58, 1.00) | 0.83 (0.58, 1.00) | 0.83 (0.58, 1.00) |
|  | 2 | 35 | 17 | 29.0 (21.0, NA) | 1.00 (1.00, 1.00) | 0.88 (0.77, 1.00) | 0.52 (0.35, 0.78) | 0.20 (0.07, 0.52) |
|  | 3 | 18 | 12 | 17.1 (13.0, NA) | 1.00 (1.00, 1.00) | 0.75 (0.56, 1.00) | 0.27 (0.12, 0.63) | 0.20 (0.07, 0.56) |
|  | 4+ | 38 | 30 | 15.6 (10.5, 27.5) | 0.78 (0.66, 0.93) | 0.56 (0.42, 0.75) | 0.35 (0.22, 0.55) | 0.08 (0.02, 0.30) |
| HR− | 1 | 12 | 7 | 27.0 (10.2, NA) | 0.82 (0.62, 1.00) | 0.63 (0.40, 1.00) | 0.50 (0.27, 0.95) | 0.19 (0.04, 0.96) |
|  | 2 | 18 | 13 | 17.1 (10.1, NA) | 1.00 (1.00, 1.00) | 0.61 (0.40, 0.91) | 0.46 (0.26, 0.81) | 0.00^3^ |
|  | 3 | 11 | 10 | 9.7 (6.4, NA) | 0.80 (0.59, 1.00) | 0.10 (0.02, 0.64) | 0.00^2^ | 0.00^3^ |
|  | 4+ | 13 | 10 | 5.8 (3.2, NA) | 0.50 (0.28, 0.89) | 0.30 (0.12, 0.75) | 0.20 (0.06, 0.68) | 0.10 (0.02, 0.63) |

*CI* confidence interval, *HR* hormone receptor, NA not available

^1^Median survival time has not been reached

^2^No patient reached an observation time of 24 months

^3^No patient reached an observation time of 60 months

**Table S3**: Mutation status with corresponding frequencies and percentages (N = 152 patients)

|  | ***BRCA1***  **N (%)** | ***BRCA2***  **N (%)** | ***PALB2***  **N (%)** |
| --- | --- | --- | --- |
| Mutated (germline) | 35 (26.1) | 61 (46.6) | 1 (0.9) |
| Wildtype (germline) | 73 (54.5) | 39 (29.8) | 32 (27.8) |
| Not tested | 26 (19.4) | 31 (23.7) | 82 (71.3) |
| Missing | 18 | 21 | 37 |

*BRCA1* breast cancer 1, *BRCA2* breast cancer 2, *PALB2* partner and localizer of *BRCA2*

**Table S4**: Frequency table of patients with a Breast Cancer Gene 1 (*BRCA1*) specified by Human Genome Variation Society (HGVS) nomenclature, showing frequency and percentage (N = 35 patients)

| ***BRCA1* mutations HGVS nomenclature** | **N (%)** |
| --- | --- |
| NC_000017.10:g.(41201212_41203079)_(41203135_41209068)del | 1 (3.8) |
| NM_007294.4(BRCA1):c.1874_1877dup (p.Val627fs) | 1 (3.8) |
| NM_007294.4(BRCA1):c.2864C>A (p.Ser955Ter) | 1 (3.8) |
| NM_007294.4(BRCA1):c.3481_3491del (p.Glu1161fs) | 2 (7.7) |
| NM_007294.4(BRCA1):c.3661G>T (p.Glu1221Ter) | 1 (3.8) |
| NM_007294.4(BRCA1):c.4183C>T (p.Gln1395Ter) | 1 (3.8) |
| NM_007294.4(BRCA1):c.4222C>T (p.Gln1408Ter) | 1 (3.8) |
| NM_007294.4(BRCA1):c.4782del (p.Ser1595fs) | 1 (3.8) |
| NM_007294.4(BRCA1):c.4986+3G>C | 1 (3.8) |
| NM_007294.4(BRCA1):c.5096G>A (p.Arg1699Gln) | 1 (3.8) |
| NM_007294.4(BRCA1):c.5266dup (p.Gln1756fs) | 1 (3.8) |
| NM_007294.4(BRCA1):c.5267A>C (p.Gln1756Pro) | 1 (3.8) |
| Unknown | 13 (50.0) |
| Missing | 9 |

**Table S5**: Frequency table of patients with a Breast Cancer Gene 2 (*BRCA2*) specified by Human Genome Variation Society (HGVS) nomenclature, showing frequency and percentage (N = 61 patients)

| ***BRCA2* mutations HGVS nomenclature** | **N (%)** |
| --- | --- |
| NM_000059.3(BRCA2):c.2808_2811del (p.Ala938Profs) | 3 (6.1) |
| NM_000059.4(BRCA2):c.1813dup (p.Ile605fs) | 1 (2.0) |
| NM_000059.4(BRCA2):c.2812_2815del (p.Ala938fs) | 1 (2.0) |
| NM_000059.4(BRCA2):c.3109C>T (p.Gln1037Ter) | 1 (2.0) |
| NM_000059.4(BRCA2):c.3860del (p.Asn1287fs) | 1 (2.0) |
| NM_000059.4(BRCA2):c.475+1G>A | 1 (2.0) |
| NM_000059.4(BRCA2):c.4780del (p.Met1594fs) | 1 (2.0) |
| NM_000059.4(BRCA2):c.5238dup (p.Asn1747Ter) | 1 (2.0) |
| NM_000059.4(BRCA2):c.5350_5351del (p.Asn1784fs) | 1 (2.0) |
| NM_000059.4(BRCA2):c.5645C>A (p.Ser1882Ter) | 1 (2.0) |
| NM_000059.4(BRCA2):c.5864C>A (p.Ser1955Ter) | 1 (2.0) |
| NM_000059.4(BRCA2):c.5946del (p.Ser1982fs) | 1 (2.0) |
| NM_000059.4(BRCA2):c.6025C>T (p.Gln2009Ter) | 1 (2.0) |
| NM_000059.4(BRCA2):c.6037A>T (p.Lys2013Ter) | 1 (2.0) |
| NM_000059.4(BRCA2):c.6275_6276del (p.Leu2092fs) | 1 (2.0) |
| NM_000059.4(BRCA2):c.6275T>C (p.Leu2092Pro) | 1 (2.0) |
| NM_000059.4(BRCA2):c.631+1G>A | 1 (2.0) |
| NM_000059.4(BRCA2):c.6447_6448del (p.Lys2150fs) | 1 (2.0) |
| NM_000059.4(BRCA2):c.67+1G>T | 1 (2.0) |
| NM_000059.4(BRCA2):c.7543del (p.Thr2515fs) | 1 (2.0) |
| NM_000059.4(BRCA2):c.8246AGA[1] (p.Lys2750del) | 1 (2.0) |
| NM_000059.4(BRCA2):c.8363G>A (p.Trp2788Ter) | 1 (2.0) |
| NM_000059.4(BRCA2):c.8633-2A>G | 1 (2.0) |
| NM_000059.4(BRCA2):c.8695C>T (p.Gln2899Ter) | 1 (2.0) |
| NM_000059.4(BRCA2):c.8755-1G>A | 1 (2.0) |
| NM_000059.4(BRCA2):c.9097del (p.Thr3033fs) | 1 (2.0) |
| NM_000059.4(BRCA2):c.9154C>T (p.Arg3052Trp) | 1 (2.0) |
| NM_000059.4(BRCA2):c.9371A>T (p.Asn3124Ile) | 2 (4.1) |
| NM_000059.4(BRCA2):c.9376del (p.Gln3126fs) | 1 (2.0) |
| NM_000059.4(BRCA2):c.9581C>A (p.Pro3194Gln) | 1 (2.0) |
| Unknown | 16 (32.7) |
| Missing | 12 |

**Table S6**: Frequency table of patients with a Breast Cancer Gene 1 (*BRCA1*) mutation classified by clinical variation (ClinVar) classification, showing frequency and percentage (N = 35 patients)

| ***BRCA1* mutation clinvar classification** | **N (%)** |
| --- | --- |
| Pathogenic | 17 (65.4) |
| Pathogenic/likely pathogenic | 1 (3.8) |
| Uncertain significance | 1 (3.8) |
| Unknown | 7 (26.9) |
| Missing | 9 |

**Table S7**: Frequency table of patients with a Breast Cancer Gene 2 (*BRCA2*) mutation classified by clinical variation (ClinVar) classification, showing frequency and percentage (N = 61 patients)

| ***BRCA2* mutation clinvar classification** | **N (%)** |
| --- | --- |
| Pathogenic | 35 (71.4) |
| Uncertain significance | 4 (8.2) |
| Unknown | 10 (20.4) |
| Missing | 12 |

**Table S8**. Summary of all adverse events (AEs) by Medical Dictionary for Regulatory Activities (MedDRA) system organ class (SOC) and preferred term (PT). Percentages refer to the total numbers of events (N = 209 events) and patients (N = 152 patients)^1^

| **MedDRA SOC** | **MedDRA PT** | **Events N (%)** | **Patients N (%)** |
| --- | --- | --- | --- |
| Blood and lymphatic system disorders | Anemia | 7 (3.3) | 7 (4.6) |
| Blood and lymphatic system disorders | Blood and lymphatic system disorders - Other, specify | 8 (3.8) | 8 (5.3) |
| Cardiac disorders | Chest pain - cardiac | 1 (0.5) | 1 (0.7) |
| Cardiac disorders | Pericardial effusion | 1 (0.5) | 1 (0.7) |
| Cardiac disorders | Supraventricular tachycardia | 2 (1.0) | 2 (1.3) |
| Eye disorders | Retinal detachment | 1 (0.5) | 1 (0.7) |
| Gastrointestinal disorders | Abdominal pain | 3 (1.4) | 2 (1.3) |
| Gastrointestinal disorders | Ascites | 1 (0.5) | 1 (0.7) |
| Gastrointestinal disorders | Constipation | 1 (0.5) | 1 (0.7) |
| Gastrointestinal disorders | Diarrhea | 8 (3.8) | 6 (3.9) |
| Gastrointestinal disorders | Dry mouth | 1 (0.5) | 1 (0.7) |
| Gastrointestinal disorders | Dyspepsia | 1 (0.5) | 1 (0.7) |
| Gastrointestinal disorders | Gastritis | 1 (0.5) | 1 (0.7) |
| Gastrointestinal disorders | Gastroesophageal reflux disease | 2 (1.0) | 2 (1.3) |
| Gastrointestinal disorders | Mucositis oral | 4 (1.9) | 4 (2.6) |
| Gastrointestinal disorders | Nausea | 11 (5.3) | 11 (7.2) |
| Gastrointestinal disorders | Toothache | 1 (0.5) | 1 (0.7) |
| Gastrointestinal disorders | Vomiting | 1 (0.5) | 1 (0.7) |
| General disorders and administration site conditions | Chills | 1 (0.5) | 1 (0.7) |
| General disorders and administration site conditions | Disease progression | 13 (6.2) | 9 (5.9) |
| General disorders and administration site conditions | Edema limbs | 1 (0.5) | 1 (0.7) |
| General disorders and administration site conditions | Fatigue | 13 (6.2) | 13 (8.6) |
| General disorders and administration site conditions | Fever | 3 (1.4) | 2 (1.3) |
| General disorders and administration site conditions | General disorders and administration site conditions - Other, specify | 6 (2.9) | 5 (3.3) |
| Hepatobiliary disorders | Hepatic failure | 2 (1.0) | 2 (1.3) |
| Immune system disorders | Allergic reaction | 2 (1.0) | 2 (1.3) |
| Infections and infestations | Breast infection | 1 (0.5) | 1 (0.7) |
| Infections and infestations | Gallbladder infection | 1 (0.5) | 1 (0.7) |
| Infections and infestations | Herpes simplex reactivation | 1 (0.5) | 1 (0.7) |
| Infections and infestations | Lung infection | 3 (1.4) | 3 (2.0) |
| Infections and infestations | Skin infection | 1 (0.5) | 1 (0.7) |
| Infections and infestations | Upper respiratory infection | 1 (0.5) | 1 (0.7) |
| Infections and infestations | Urinary tract infection | 3 (1.4) | 3 (2.0) |
| Injury, poisoning and procedural complications | Burn | 1 (0.5) | 1 (0.7) |
| Investigations | Blood bilirubin increased | 1 (0.5) | 1 (0.7) |
| Metabolism and nutrition disorders | Anorexia | 3 (1.4) | 3 (2.0) |
| Metabolism and nutrition disorders | Hyperglycemia | 1 (0.5) | 1 (0.7) |
| Metabolism and nutrition disorders | Hypokalemia | 1 (0.5) | 1 (0.7) |
| Musculoskeletal and connective tissue disorders | Arthralgia | 2 (1.0) | 2 (1.3) |
| Musculoskeletal and connective tissue disorders | Back pain | 3 (1.4) | 3 (2.0) |
| Musculoskeletal and connective tissue disorders | Chest wall pain | 1 (0.5) | 1 (0.7) |
| Musculoskeletal and connective tissue disorders | Flank pain | 1 (0.5) | 1 (0.7) |
| Musculoskeletal and connective tissue disorders | Joint effusion | 1 (0.5) | 1 (0.7) |
| Musculoskeletal and connective tissue disorders | Joint range of motion decreased lumbar spine | 1 (0.5) | 1 (0.7) |
| Musculoskeletal and connective tissue disorders | Muscle cramp | 2 (1.0) | 2 (1.3) |
| Musculoskeletal and connective tissue disorders | Myalgia | 1 (0.5) | 1 (0.7) |
| Musculoskeletal and connective tissue disorders | Pain in extremity | 11 (5.3) | 8 (5.3) |
| Nervous system disorders | Concentration impairment | 1 (0.5) | 1 (0.7) |
| Nervous system disorders | Dizziness | 1 (0.5) | 1 (0.7) |
| Nervous system disorders | Dysarthria | 2 (1.0) | 2 (1.3) |
| Nervous system disorders | Dysgeusia | 2 (1.0) | 2 (1.3) |
| Nervous system disorders | Extrapyramidal disorder | 2 (1.0) | 2 (1.3) |
| Nervous system disorders | Headache | 7 (3.3) | 4 (2.6) |
| Nervous system disorders | Peripheral motor neuropathy | 1 (0.5) | 1 (0.7) |
| Nervous system disorders | Peripheral sensory neuropathy | 5 (2.4) | 5 (3.3) |
| Nervous system disorders | Seizure | 1 (0.5) | 1 (0.7) |
| Psychiatric disorders | Insomnia | 1 (0.5) | 1 (0.7) |
| Renal and urinary disorders | Urinary tract obstruction | 7 (3.3) | 2 (1.3) |
| Reproductive system and breast disorders | Breast pain | 1 (0.5) | 1 (0.7) |
| Reproductive system and breast disorders | Pelvic pain | 1 (0.5) | 1 (0.7) |
| Respiratory, thoracic and mediastinal disorders | Cough | 2 (1.0) | 2 (1.3) |
| Respiratory, thoracic and mediastinal disorders | Dyspnea | 7 (3.3) | 6 (3.9) |
| Respiratory, thoracic and mediastinal disorders | Pleural effusion | 6 (2.9) | 5 (3.3) |
| Skin and subcutaneous tissue disorders | Alopecia | 6 (2.9) | 6 (3.9) |
| Skin and subcutaneous tissue disorders | Dry skin | 4 (1.9) | 4 (2.6) |
| Skin and subcutaneous tissue disorders | Hyperhidrosis | 2 (1.0) | 2 (1.3) |
| Skin and subcutaneous tissue disorders | Nail changes | 2 (1.0) | 2 (1.3) |
| Skin and subcutaneous tissue disorders | Pruritus | 1 (0.5) | 1 (0.7) |
| Skin and subcutaneous tissue disorders | Rash maculo-papular | 1 (0.5) | 1 (0.7) |
| Skin and subcutaneous tissue disorders | Skin and subcutaneous tissue disorders - Other, specify | 1 (0.5) | 1 (0.7) |
| Vascular disorders | Hot flashes | 4 (1.9) | 4 (2.6) |
| Vascular disorders | Hypertension | 4 (1.9) | 3 (2.0) |

^1^ The table can be read as follows: For example, 13 disease progression events were observed and these events occurred in 9 patients. In other words, there were 9 patients with one or more disease progression events.

**Table S9**. Summary of adverse events (AEs) of grade 3 to 5 by Medical Dictionary for Regulatory Activities (MedDRA) system organ class (SOC) and preferred term (PT). Percentages refer to the total numbers of events (N = 42 events) and patients (N = 152 patients)^1^

| **MedDRA SOC** | **MedDRA PT** | **Events N (%)** | **Patients N (%)** |
| --- | --- | --- | --- |
| Blood and lymphatic system disorders | Anemia | 2 (4.8) | 2 (1.3) |
| Blood and lymphatic system disorders | Blood and lymphatic system disorders - Other, specify | 3 (7.1) | 3 (2.0) |
| Eye disorders | Retinal detachment | 1 (2.4) | 1 (0.7) |
| Gastrointestinal disorders | Ascites | 1 (2.4) | 1 (0.7) |
| General disorders and administration site conditions | Disease progression | 10 (23.8) | 8 (5.3) |
| General disorders and administration site conditions | Fever | 1 (2.4) | 1 (0.7) |
| General disorders and administration site conditions | General disorders and administration site conditions - Other, specify | 6 (14.3) | 5 (3.3) |
| Infections and infestations | Gallbladder infection | 1 (2.4) | 1 (0.7) |
| Infections and infestations | Lung infection | 1 (2.4) | 1 (0.7) |
| Infections and infestations | Skin infection | 1 (2.4) | 1 (0.7) |
| Metabolism and nutrition disorders | Hyperglycemia | 1 (2.4) | 1 (0.7) |
| Musculoskeletal and connective tissue disorders | Back pain | 2 (4.8) | 2 (1.3) |
| Musculoskeletal and connective tissue disorders | Pain in extremity | 1 (2.4) | 1 (0.7) |
| Nervous system disorders | Dysarthria | 1 (2.4) | 1 (0.7) |
| Nervous system disorders | Dysgeusia | 1 (2.4) | 1 (0.7) |
| Nervous system disorders | Headache | 1 (2.4) | 1 (0.7) |
| Nervous system disorders | Peripheral sensory neuropathy | 1 (2.4) | 1 (0.7) |
| Nervous system disorders | Seizure | 1 (2.4) | 1 (0.7) |
| Renal and urinary disorders | Urinary tract obstruction | 1 (2.4) | 1 (0.7) |
| Respiratory, thoracic and mediastinal disorders | Dyspnea | 2 (4.8) | 2 (1.3) |
| Respiratory, thoracic and mediastinal disorders | Pleural effusion | 3 (7.1) | 3 (2.0) |

^1^ The table can be read as follows: For example, 10 disease progression events were observed and these events occurred in 8 patients. In other words, there were 8 patients with one or more disease progression events.

**Table S10**. Summary of all serious adverse events (SAEs) by Medical Dictionary for Regulatory Activities (MedDRA) system organ class (SOC) and preferred term (PT). Percentages refer to the total numbers of events (N = 41 events) and patients (N = 152 patients)^1^

| **MedDRA SOC** | **MedDRA PT** | **Events N (%)** | **Patients N (%)** |
| --- | --- | --- | --- |
| Blood and lymphatic system disorders | Anemia | 2 (4.9) | 2 (1.3) |
| Gastrointestinal disorders | Ascites | 1 (2.4) | 1 (0.7) |
| Gastrointestinal disorders | Nausea | 1 (2.4) | 1 (0.7) |
| General disorders and administration site conditions | Disease progression | 12 (29.3) | 9 (5.9) |
| General disorders and administration site conditions | Fever | 1 (2.4) | 1 (0.7) |
| General disorders and administration site conditions | General disorders and administration site conditions - Other, specify | 6 (14.6) | 5 (3.3) |
| Hepatobiliary disorders | Hepatic failure | 1 (2.4) | 1 (0.7) |
| Infections and infestations | Breast infection | 1 (2.4) | 1 (0.7) |
| Infections and infestations | Gallbladder infection | 1 (2.4) | 1 (0.7) |
| Infections and infestations | Lung infection | 2 (4.9) | 2 (1.3) |
| Infections and infestations | Skin infection | 1 (2.4) | 1 (0.7) |
| Investigations | Blood bilirubin increased | 1 (2.4) | 1 (0.7) |
| Metabolism and nutrition disorders | Hyperglycemia | 1 (2.4) | 1 (0.7) |
| Musculoskeletal and connective tissue disorders | Back pain | 1 (2.4) | 1 (0.7) |
| Musculoskeletal and connective tissue disorders | Pain in extremity | 1 (2.4) | 1 (0.7) |
| Nervous system disorders | Seizure | 1 (2.4) | 1 (0.7) |
| Renal and urinary disorders | Urinary tract obstruction | 1 (2.4) | 1 (0.7) |
| Respiratory, thoracic and mediastinal disorders | Dyspnea | 2 (4.9) | 2 (1.3) |
| Respiratory, thoracic and mediastinal disorders | Pleural effusion | 4 (9.8) | 3 (2.0) |

^1^ The table can be read as follows: For example, 12 disease progression events were observed and these events occurred in 9 patients. In other words, there were 9 patients with one or more disease progression events.

**Table S11.** STROBE Statement—checklist of items that should be included in reports of observational studies [1]

|  | | Item No. | Recommendation | | | Page  No. | | | Relevant text from manuscript |  |
| --- | --- | --- | --- | --- | --- | --- | --- | --- | --- | --- |
| **Title and abstract** | | 1 | (*a*) Indicate the study’s design with a commonly used term in the title or the abstract | | | 1 | | PARP Inhibition with Olaparib and Talazoparib for HER2-negative Advanced Breast Cancer—Results from the Prospective PRAEGNANT Registry | |  |
|  |  |  | (*b*) Provide in the abstract an informative and balanced summary of what was done and what was found | | | 5 | | We provide a real-world analysis for PARP-inhibitor use in ABC patients treated within the prospective German PRAEGNANT registry (NCT02338167). 152 patients with ABC receiving a PARP-inhibitor were included. Real-world progression-free survival (rwPFS) and real-world overall survival (rwOS) were calculated for all patients using the Kaplan–Meier method. | |  |
| Introduction | | | | | | | | |  |  |
| Background/rationale | | 2 | Explain the scientific background and rationale for the investigation being reported | | | 6 | | … In the OlympiAD trial, patients treated with olaparib had a median progression-free survival (PFS) of 7.0 (95% confidence interval [CI], 0.43–0.80) months and an overall survival (OS) of 19.3 months compared to 4.2 months (hazard ratio 0.58; 95% confidence interval [CI], 0.43–0.80) and 17.1 (hazard ratio 0.90, 95%-CI 0.66-1.23) months in the control arm for patients receiving chemotherapy of physician’s choice (capecitabine, eribulin, vinorelbine)… | |  |
| Objectives | | 3 | State specific objectives, including any prespecified hypotheses | | | 6 | | Few prospective real-world analyses with PARP-inhibitors have been published and treatment reality in Germany regarding PARP-inhibitor therapy for ABC remains unknown. This analysis, therefore, aims to shed light on the treatment reality with PARP-inhibitors in patients included into the prospective German PRAEGNANT registry, with a focus on subgroups, germline mutations and adverse events. | |  |
| Methods | | | | | | | | |  |  |
| Study design | | 4 | Present key elements of study design early in the paper | | | 20 | | The PRAEGNANT … study is an ongoing, prospective breast cancer registry. Documentation is similar to that of a clinical trial. The first patient was recruited in July 2014. The PRAEGNANT registry aims to assess treatment patterns, to investigate quality of life and survivorship, … | |  |
| Setting | | 5 | Describe the setting, locations, and relevant dates, including periods of recruitment, exposure, follow-up, and data collection | | | 20 | | Patients can be included at any given point during the course of their disease. Follow-up assessments for the advanced setting are updated every three months until month 24 and thereafter every six months in case there is no progression or change of therapy within three months of observation. Furthermore, biomaterials from blood and tumor biopsies are collected for research purposes | |  |
| Participants | | 6 | (*a*) *Cohort study*—Give the eligibility criteria, and the sources and methods of selection of participants. Describe methods of follow-up | | | 20 | | At the time of data cut-off (October 07, 2025), 6,402 ABC patients were registered in the PRAEGNANT registry. Of these, 5,893 patients had both documented HR and HER2 status. Of this overall population, 1,138 patients were excluded due to HER2-positive disease, leaving 4,755 patients… | |  |
| Variables | | 7 | Clearly define all outcomes, exposures, predictors, potential confounders, and effect modifiers. Give diagnostic criteria, if applicable | | | 22 | | The primary objective was to investigate rwPFS in patients receiving olaparib or talazoparib. Survival rates with 95%-CIs and median survival times were estimated using the Kaplan-Meier product-limit method. | |  |
| Data sources/ measurement | | 8* | For each variable of interest, give sources of data and details of methods of assessment (measurement). Describe comparability of assessment methods if there is more than one group | | | *22* | | Data was collected by trained personnel and documented in an electronic case report form. Automated plausibility checks issued and addressed queries regarding the research question, and on-site monitoring was performed…. | |  |
| Bias | | 9 | Describe any efforts to address potential sources of bias | | | 18-19 | | The adverse events described in our cohort are similar to those reported in the pivotal trials but were reported at a much lower frequency. Ascertainment bias likely resulted in and under-reporting in our real-world trial similar to other real-world studies. | |  |
| Study size | | 10 | Explain how the study size was arrived at | | | 20 | | … Consequently, 152 patients were included in the final patient population. The patient flow chart is shown in Figure 4. | |  |
| Quantitative variables | 11 | | | Explain how quantitative variables were handled in the analyses. If applicable, describe which groupings were chosen and why | 22 | | Mean and standard deviation and median and interquartile range (IQR) are calculated for continuous variables, whereas frequencies and percentages are used for categorical variables. | | | |
| Statistical methods | 12 | | | (*a*) Describe all statistical methods, including those used to control for confounding | 22 | | Real-world PFS (rwPFS) was defined as the time from the start of therapy to the earliest occurrence of disease progression (distant-metastasis, local recurrence, or death from any cause), or the last known date the patient was progression-free… Real-world OS (rwOS) was defined in a similar manner. | | | |
|  |  |  |  | (*b*) Describe any methods used to examine subgroups and interactions | 22 | | Subgroup analyses were conducted based on the following variables: age (categorical; up to and including 49 years, 50 to 64 years, 65+ years), body mass index… | | | |
|  |  |  |  | (*c*) Explain how missing data were addressed | 19, 20 | | Missing mutation information data must be noted as a limitation, however….  A total of 286 patients had to be excluded due to missing documentation of the date of first metastasis or unknown year of birth, 34 patients had no documented therapy, leaving 4,435 patients with documented therapies. | | | |
|  |  |  |  | (*d*) *Cohort study*—If applicable, explain how loss to follow-up was addressed | 20, 22 | | Six further patients had invalid PFS or OS follow-up data and were thus excluded…  Patients without an event were censored at the last follow-up or at 60 months (five years), whichever came first. | | | |
|  |  |  |  | (*e*) Describe any sensitivity analyses | NA | | NA | | | |

| Results | | | | |  |
| --- | --- | --- | --- | --- | --- |
| Participants | 13* | (a) Report numbers of individuals at each stage of study—eg numbers potentially eligible, examined for eligibility, confirmed eligible, included in the study, completing follow-up, and analysed | 20 | At the time of data cut-off (October 07, 2025), 6,402 ABC patients were registered in the PRAEGNANT registry. Of these, 5,893 patients had both documented HR and HER2 status. Of this overall population, 1,138 patients were excluded due to HER2-positive disease, leaving 4,755 patients, of whom 781 patients had triple-negative disease and 3,974 patients had HR-positive, HER2-negative disease…. |  |
|  |  | (b) Give reasons for non-participation at each stage | NA | NA |  |
|  |  | (c) Consider use of a flow diagram | 21 | Figure 4: Patient inclusion and exclusion flow chart |  |
| Descriptive data | 14 | (a) Give characteristics of study participants (eg demographic, clinical, social) and information on exposures and potential confounders | 7 | Baseline patient and tumor characteristics are shown in Table 1. |  |
|  |  | (b) Indicate number of participants with missing data for each variable of interest | 7 | Baseline patient and tumor characteristics are shown in Table 1. |  |
|  |  | (c) *Cohort study*—Summarise follow-up time (eg, average and total amount) | 7 | The median observation time for rwPFS was 6.1 months (IQR, 3.0–10.2 months) and 12.8 (IQR, 7.6–23.9) months for rwOS. |  |
| Outcome data | 15 | *Cohort study*—Report numbers of outcome events or summary measures over time | 12, 15 | Events for PFS are given in Table 2: Median real-world progression-free survival times and survival rates; Events for OS are given in Table 3: Median real-world overall survival times and survival rates |  |
| Main results | 16 | (*a*) Give unadjusted estimates and, if applicable, confounder-adjusted estimates and their precision (eg, 95% confidence interval). Make clear which confounders were adjusted for and why they were included | 8 | The median rwPFS time was 6.2 months (95%-CI, 4.8–7.9), …  No confounder-adjustement was carried out. |  |
|  |  | (*b*) Report category boundaries when continuous variables were categorized | 7 | For example, for age: Up to 49; 50 – 64; 65+ |  |
|  |  | (*c*) If relevant, consider translating estimates of relative risk into absolute risk for a meaningful time period | NA | NA |  |
| Other analyses | 17 | Report other analyses done—eg analyses of subgroups and interactions, and sensitivity analyses | 8, 13 | All remaining Kaplan-Meier curves for rwPFS based on different cofactors are shown in the supplementary information (Figure S1a-S1h) …  Six-month, 12-month, 24-month, and 60-month rwPFS rates with 95%-CI with regard to different cofactors are presented in Table 2 …  Six-month, 12-month, 24-month and 60-month rwOS rates with 95%-CI with regard to different cofactors are presented in Table 3 …  Further rwOS Kaplan–Meier graphs based on different cofactors are presented in the supplementary information (Figures S2a-S2h). | |

| Discussion | | | | |
| --- | --- | --- | --- | --- |
| Key results | 18 | Summarise key results with reference to study objectives | 18 | Our real-world analysis of PARP-inhibitors in patients with HER2-negative ABC shows a median rwPFS time of 6.2 months (95%-CI, 4.8-7.9)… |
| Limitations | 19 | Discuss limitations of the study, taking into account sources of potential bias or imprecision. Discuss both direction and magnitude of any potential bias | 19 | Our study has several limitations. One… |
| Interpretation | 20 | Give a cautious overall interpretation of results considering objectives, limitations, multiplicity of analyses, results from similar studies, and other relevant evidence | 19 | Our data confirms the results from the registrational trials of olaparib and talazoparib, showing very similar median rwPFS and rwOS times. |
| Generalisability | 21 | Discuss the generalisability (external validity) of the study results | 19 | With good tolerability, the RWD supports the recommendation for germline *BRCA1* and *BRCA2* testing in order to enable patients to receive therapy with a PARP-inhibitor. |
| Other information | |  | | |
| Funding | 22 | Give the source of funding and the role of the funders for the present study and, if applicable, for the original study on which the present article is based | 24 | Acknowledgments: This study received no funding |

[1] von Elm, E., et al., The Strengthening the Reporting of Observational Studies in Epidemiology (STROBE) statement: guidelines for reporting observational studies. Lancet, 2007. 370(9596): p. 1453–7.

**Table S12.** CONSORT 2025 checklist [2]

| **Section / Topic** | **No** | **CONSORT 2025 checklist item description** | **Reported on page no.** |
| --- | --- | --- | --- |
| **Title and abstract** | | |  |
| Title and structured abstract | 1a | Identification as a randomised trial | Not applicable (NA), as this study is not a randomized trial. |
|  | 1b | Structured summary of the trial design, methods, results, and conclusions | 5 |
| **Open science** | | |  |
| Trial registration | 2 | Name of trial registry, identifying number (with URL) and date of registration | 20 |
| Protocol and statistical analysis plan | 3 | Where the trial protocol and statistical analysis plan can be accessed | The Protocol Synopsis can be accessed publicly: https://praegnant.org/wp-content/uploads/2025/08/PRAEGNANT_Synopsis_Amendment-4_20241030.pdf |
| Data sharing | 4 | Where and how the individual de-identified participant data (including data dictionary), statistical code and any other materials can be accessed | 24 |
| Funding and conflicts of interest | 5a | Sources of funding and other support (e.g., supply of drugs), and role of funders in the design, conduct, analysis and reporting of the trial | 24 |
|  | 5b | Financial and other conflicts of interest of the manuscript authors | 24 |
| **Introduction** | | |  |
| Background and rationale | 6 | Scientific background and rationale | 6 |
| Objectives | 7 | Specific objectives related to benefits and harms | 22 |
| **Methods** | | |  |
| Patient and public involvement | 8 | Details of patient or public involvement in the design, conduct and reporting of the trial | 20 |
| Trial design | 9 | Description of trial design including type of trial (e.g., parallel group, crossover), allocation ratio, and framework (e.g., superiority, equivalence, non-inferiority, exploratory) | 20 |
| Changes to trial protocol | 10 | Important changes to the trial after it commenced including any outcomes or analyses that were not prespecified, with reason | 20 |
| Trial setting | 11 | Settings (e.g., community, hospital) and locations (e.g., countries, sites) where the trial was conducted | 20 |
| Eligibility criteria | 12a | Eligibility criteria for participants | 20 |
|  | 12b | If applicable, eligibility criteria for sites and for individuals delivering the interventions (e.g., surgeons, physiotherapists) | NA |
| Intervention and comparator | 13 | Intervention and comparator with sufficient details to allow replication. If relevant, where additional materials describing the intervention and comparator (e.g., intervention manual) can be accessed | NA |
| Outcomes | 14 | Pre-specified primary and secondary outcomes, including the specific measurement variable (e.g., systolic blood pressure), analysis metric (e.g., change from baseline, final value, time to event), method of aggregation (e.g., median, proportion), and time point for each outcome | 22 |
| Harms | 15 | How harms were defined and assessed (e.g., systematically, non-systematically) | NA; As this was a purely observational prospective study, no harm to participants was expected. |
| Sample size | 16a | How sample size was determined, including all assumptions supporting the sample size calculation | 20, As this was a purely observational prospective study, no sample size calculation was performed. |
|  | 16b | Explanation of any interim analyses and stopping guidelines | NA; As this was a purely observational prospective study, no interim analyses or stopping guidelines applied. |
| Randomisation: |  |  |  |
| Sequence generation | 17a | Who generated the random allocation sequence and the method used | NA; As this was a purely observational prospective study, no randomization was performed. |
|  | 17b | Type of randomisation and details of any restriction (e.g., stratification, blocking and block size) | NA; As this was a purely observational prospective study, no randomization was performed. |
| Allocation concealment mechanism | 18 | Mechanism used to implement the random allocation sequence (e.g., central computer/telephone; sequentially numbered, opaque, sealed containers), describing any steps to conceal the sequence until interventions were assigned | NA; As this was a purely observational prospective study, no randomization was performed. |
| Implementation | 19 | Whether the personnel who enrolled and those who assigned participants to the interventions had access to the random allocation sequence | NA; As this was a purely observational prospective study, no randomization was performed. |
| Blinding | 20a | Who was blinded after assignment to interventions (e.g., participants, care providers, outcome assessors, data analysts) | NA; As this was a purely observational prospective study, no randomization was performed and nobody had to be blinded. |
|  | 20b | If blinded, how blinding was achieved and description of the similarity of interventions | NA |
| Statistical methods | 21a | Statistical methods used to compare groups for primary and secondary outcomes, including harms | NA; As this was a purely observational prospective study there were no randomized groups to compare |
|  | 21b | Definition of who is included in each analysis (e.g., all randomised participants), and in which group | NA; As this was a purely observational prospective study there were no randomized groups to compare |
|  | 21c | How missing data were handled in the analysis | 20 |
|  | 21d | Methods for any additional analyses (e.g., subgroup and sensitivity analyses), distinguishing prespecified from post-hoc | 22, 23 |
| **Results** | | |  |
| Participant flow, including flow diagram | 22a | For each group, the numbers of participants who were randomly assigned, received intended intervention, and were analysed for the primary outcome | NA; As this was a purely observational prospective study there were no randomized groups to compare |
|  | 22b | For each group, losses and exclusions after randomisation, together with reasons | NA; As this was a purely observational prospective study there were no randomized groups to compare |
| Recruitment | 23a | Dates defining the periods of recruitment and follow-up for outcomes of benefits and harms | 20 |
|  | 23b | If relevant, why the trial ended or was stopped | NA |
| Intervention and comparator delivery | 24a | Intervention and comparator as they were actually administered (e.g., where appropriate, who delivered the intervention/comparator, how participants adhered, whether they were delivered as intended [fidelity]) | NA; As this was a purely observational prospective study evaluating the standard of care treatment. |
|  | 24b | Concomitant care received during the trial for each group | NA; As this was a purely observational prospective study evaluating the standard of care treatment. |
| Baseline data | 25 | A table showing baseline demographic and clinical characteristics for each group | 2; as this was a purely observational prospective study, there were no randomized groups to compare. |
| Numbers analysed,  outcomes and estimation | 26 | For each primary and secondary outcome, by group:   - the number of participants included in the analysis - the number of participants with available data at the outcome time point - result for each group, and the estimated effect size and its precision (such as 95% confidence interval) - for binary outcomes, presentation of both absolute and relative effect size | NA; as this was a purely observational prospective study, there were no randomized groups to compare. |
| Harms | 27 | All harms or unintended events in each group | NA; as this was a purely observational prospective study, there were no randomized groups to compare. |
| Ancillary analyses | 28 | Any other analyses performed, including subgroup and sensitivity analyses, distinguishing pre-specified from post-hoc | 7-17 |
| **Discussion** | | |  |
| Interpretation | 29 | Interpretation consistent with results, balancing benefits and harms, and considering other relevant evidence | 18 |
| Limitations | 30 | Trial limitations, addressing sources of potential bias, imprecision, generalisability, and, if relevant, multiplicity of analyses | 19 |

The checklist was downloaded from: [www.consort-spirit.org](http://www.consort-spirit.org) on March 18, 2026.

[2] Hopewell S, Chan AW, Collins GS, Hróbjartsson A, Moher D, Schulz KF, et al. CONSORT 2025 Statement: updated guideline for reporting randomised trials. BMJ. 2025; 388:e081123. <https://dx.doi.org/10.1136/bmj-2024-081123>.

**Figure S1**: Real-world progression-free survival relative to different subgroups

**Figure S1a**: Real-world progression-free survival relative to the age (years)


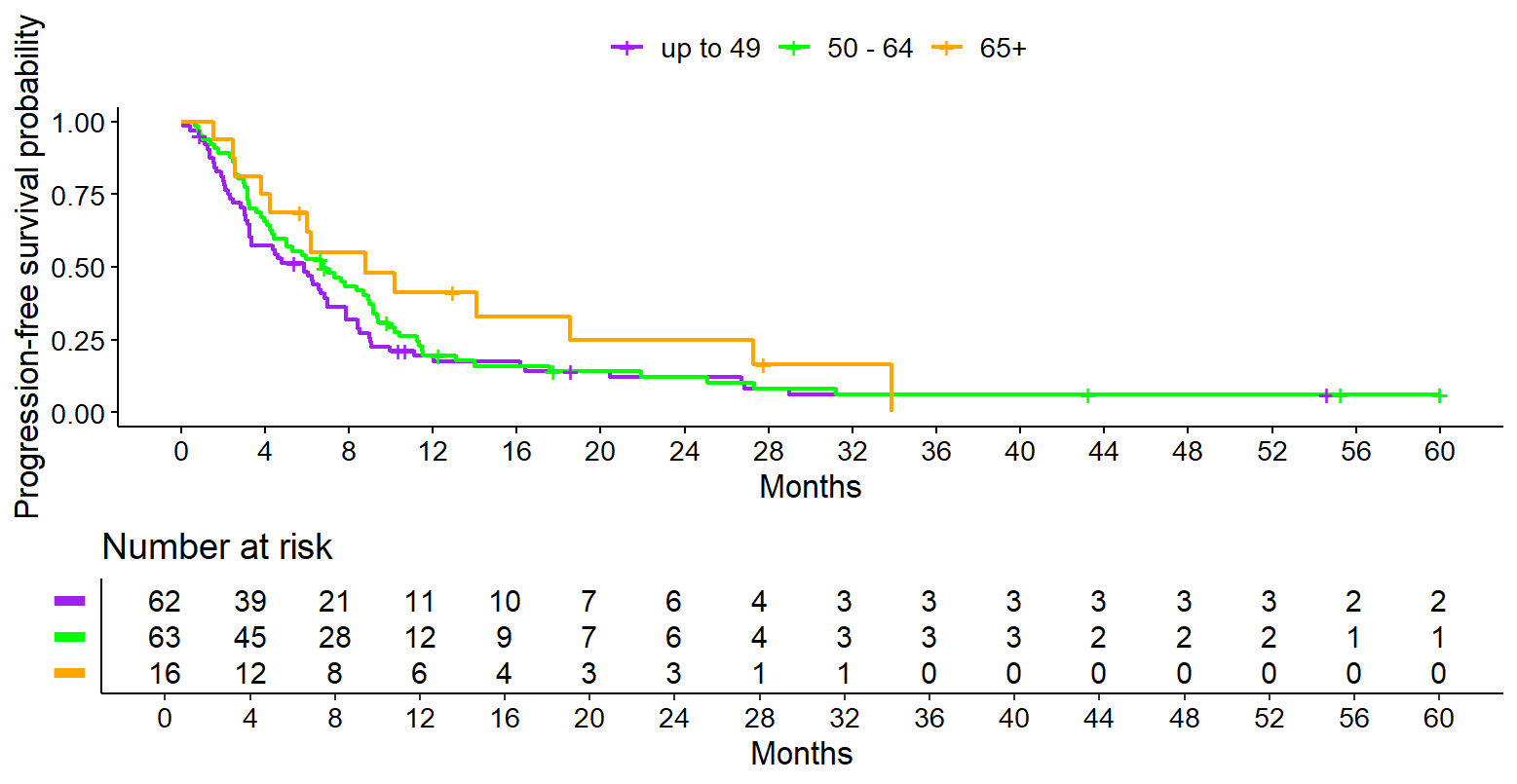


**Figure S1b**: Real-world progression-free survival relative to the body mass index


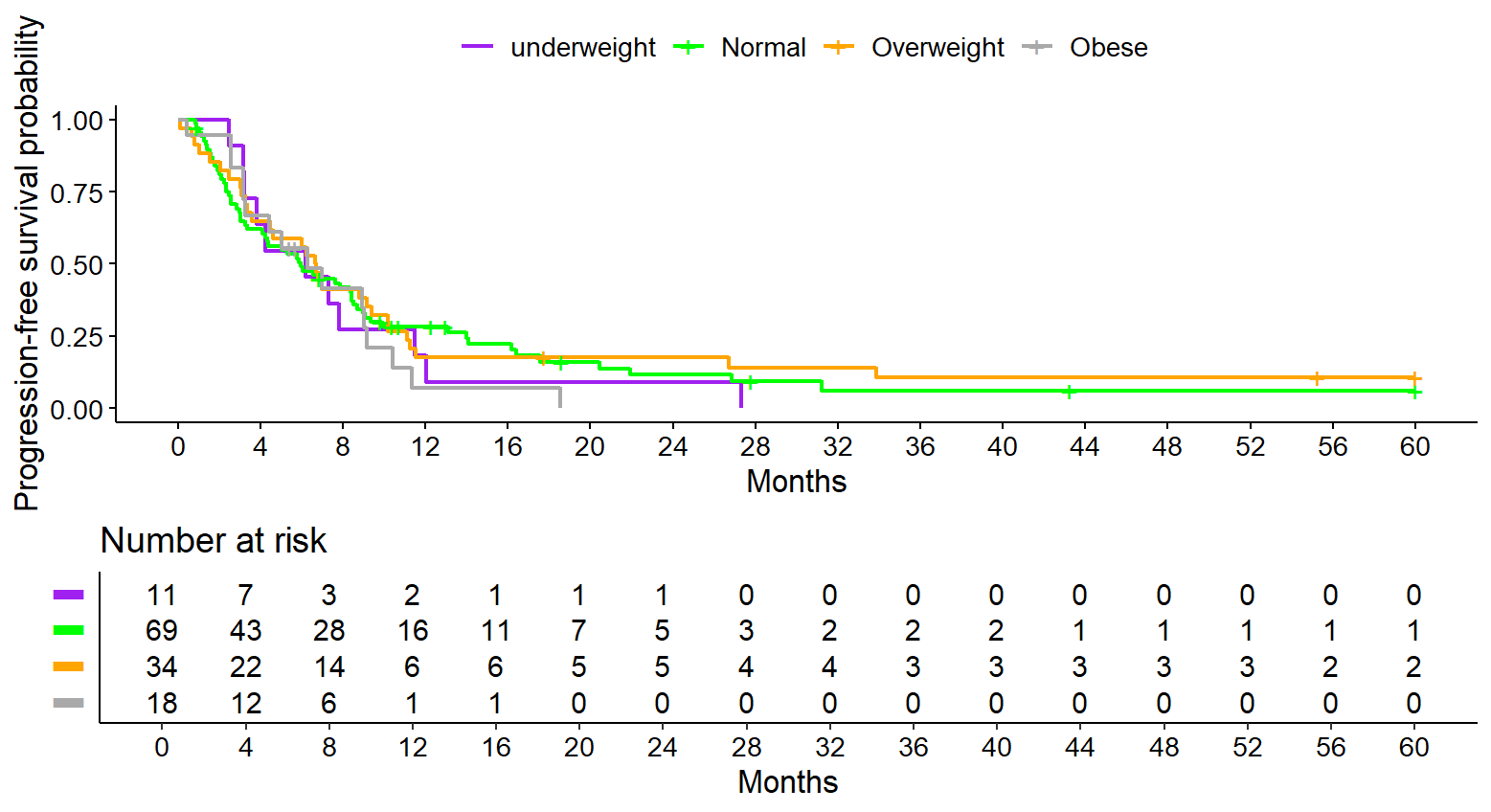


**Figure S1c**: Real-world progression-free survival relative to the Eastern Cooperative Oncology Group (ECOG) performance status


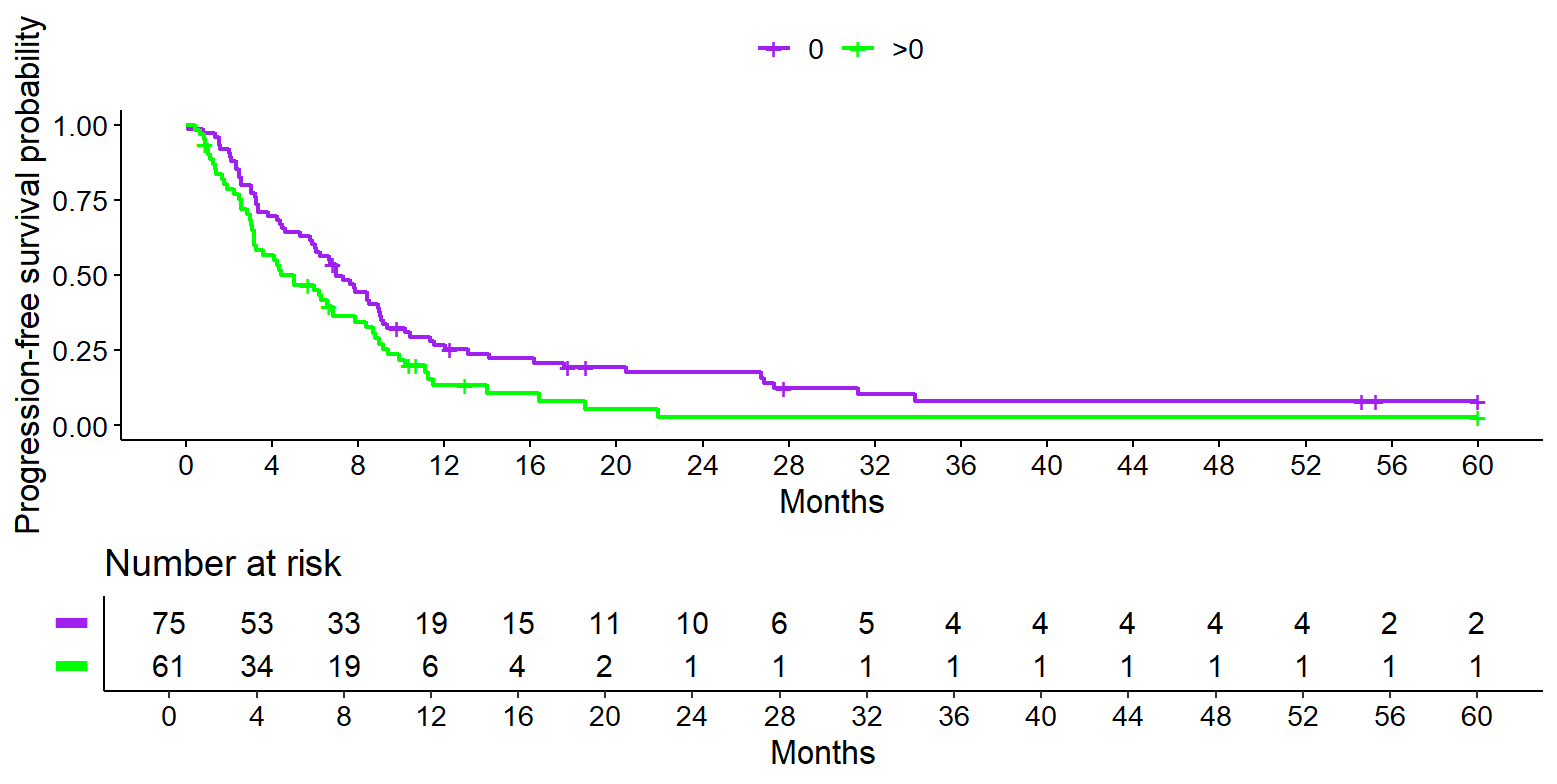


**Figure S1d**: Real-world progression-free survival relative to the tumor grading


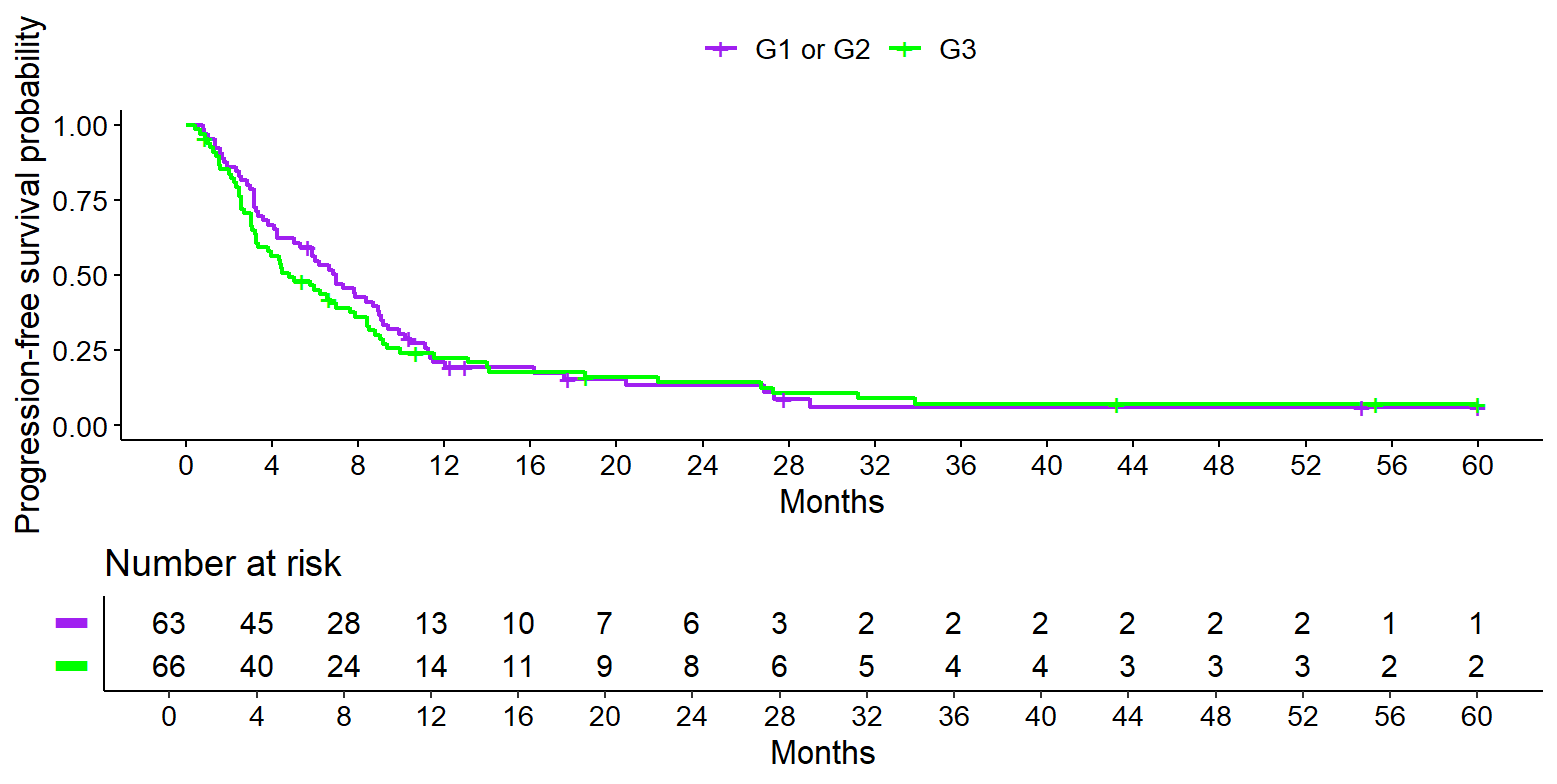


**Figure S1e**: Real-world progression-free survival of all patients relative to the line of therapy


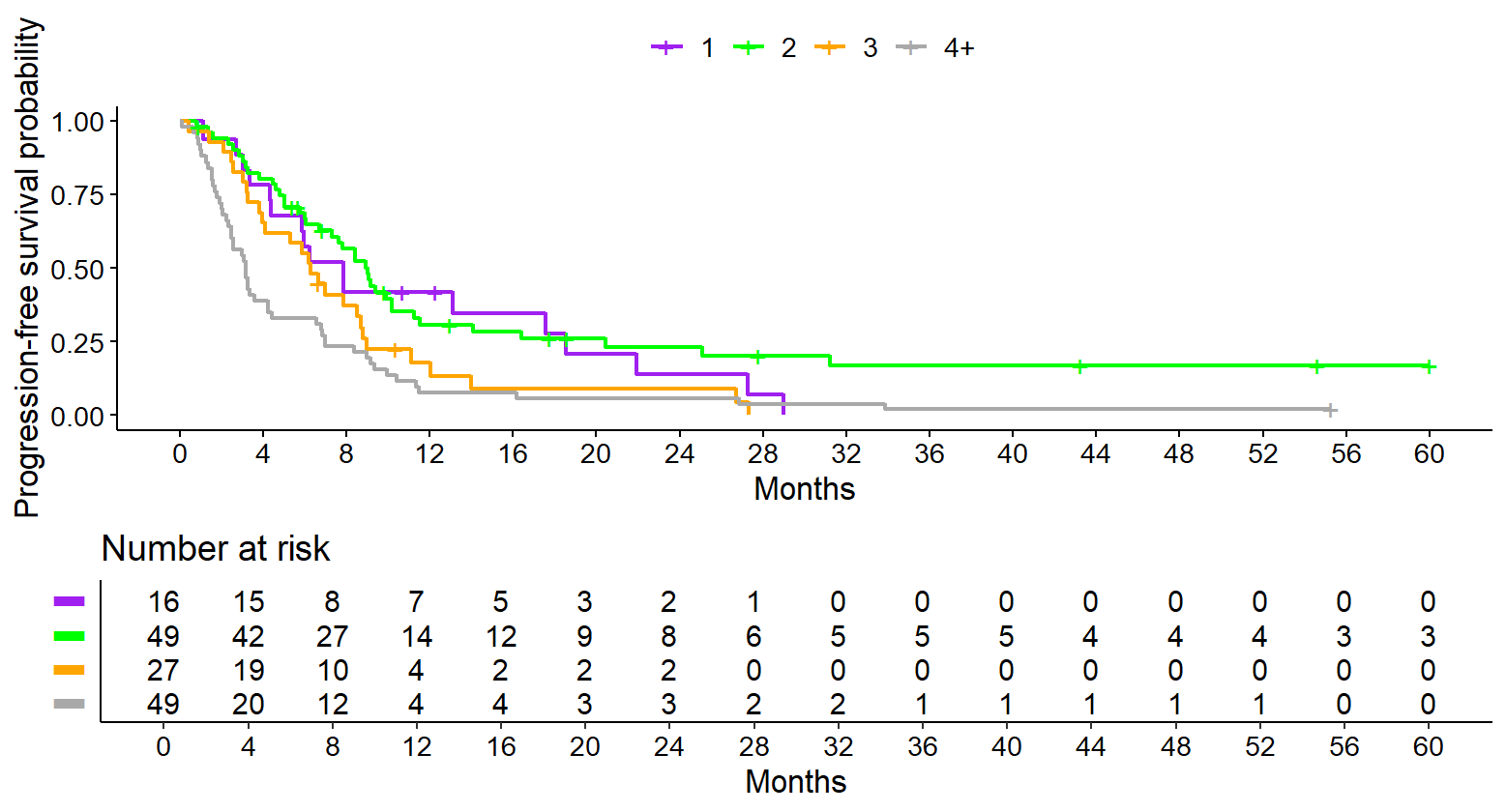


**Figure S1f**: Real-world progression-free survival relative to metastasis pattern


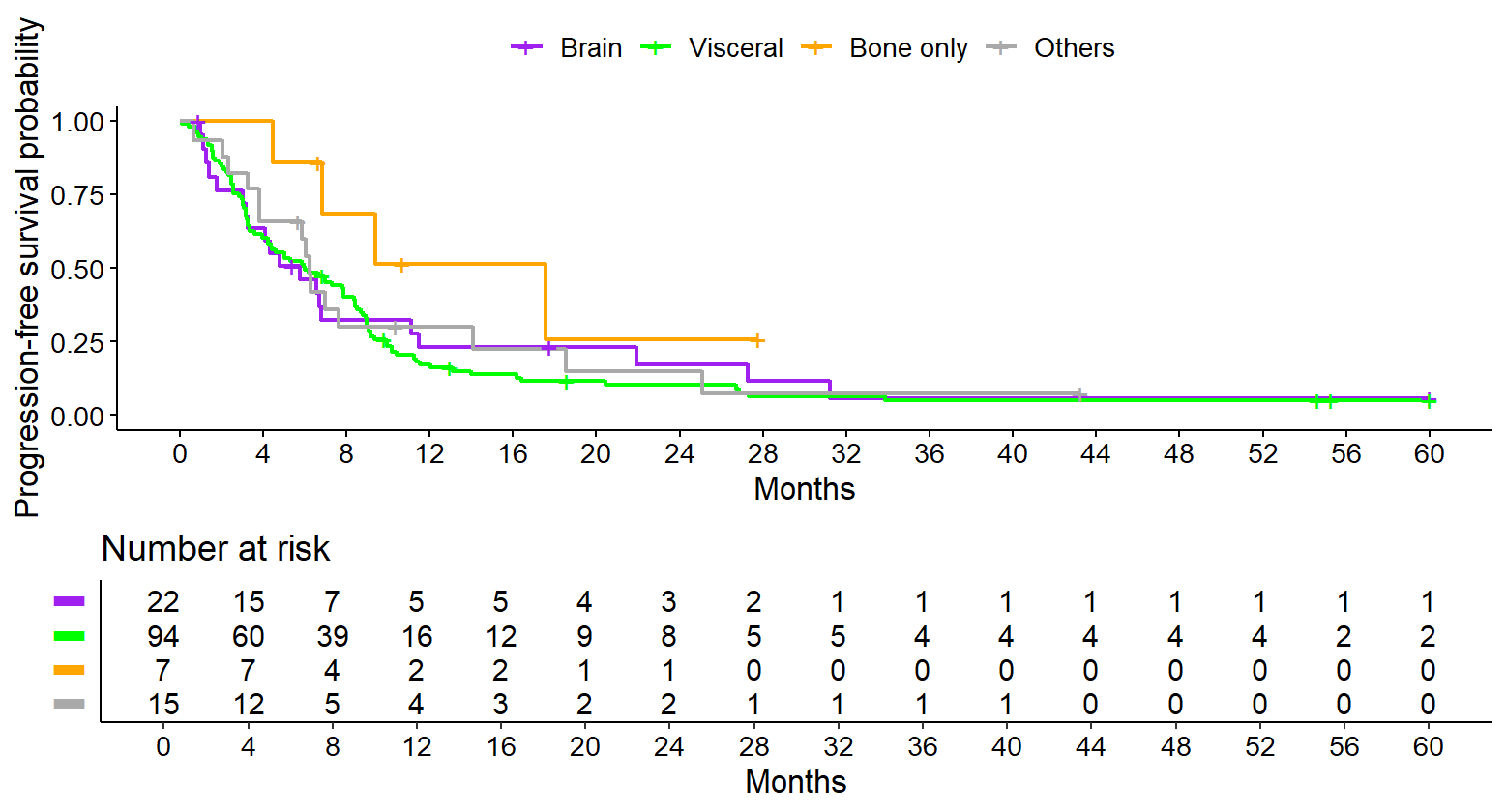


**Figure S1g**: Real-world progression-free survival relative to metastasis timing (de novo, ≤ 60 months after primary diagnosis, > 60 months after primary diagnosis)


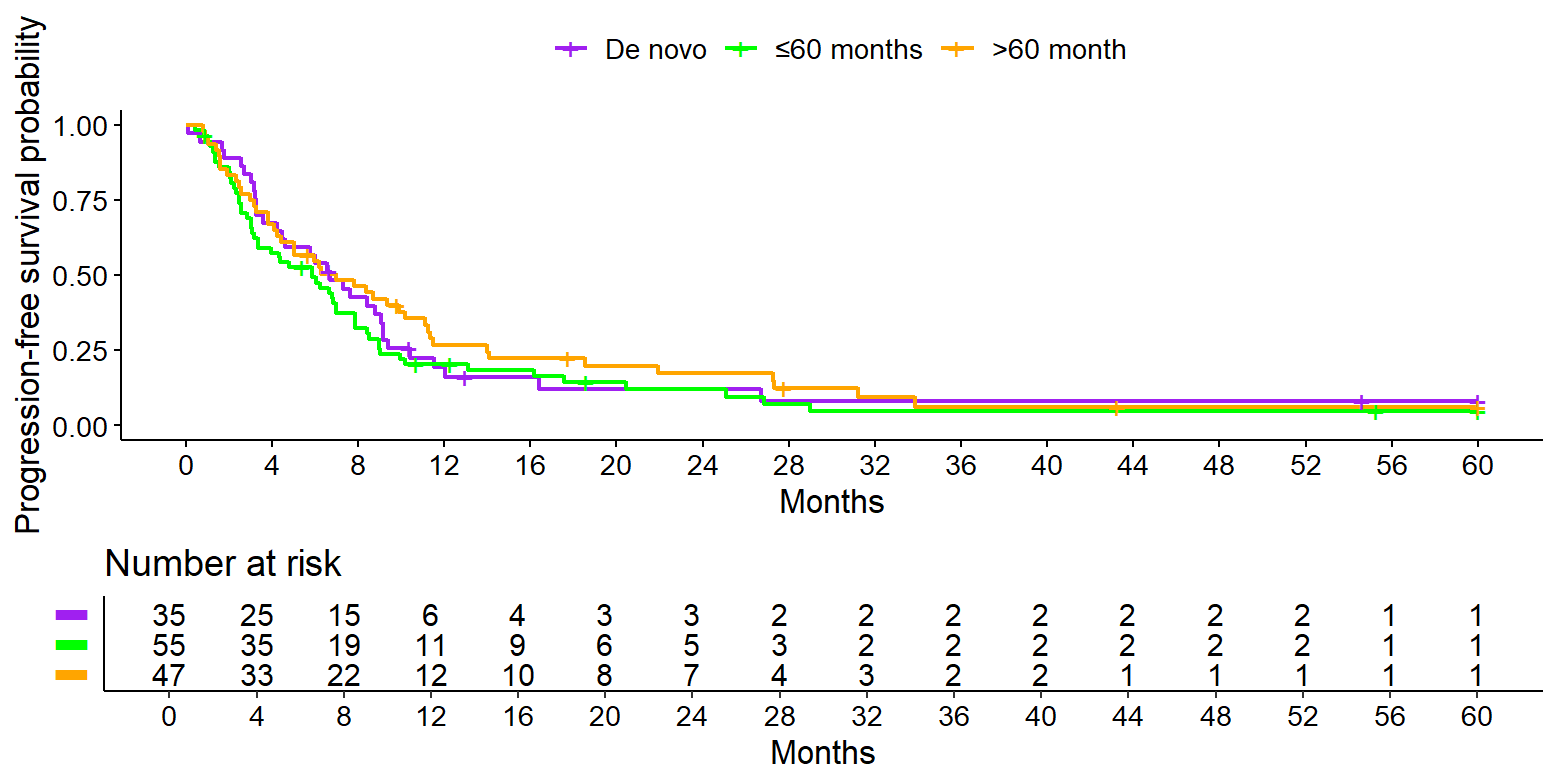


**Figure S1h**: Real-world progression-free survival relative to the number of concomitant diseases


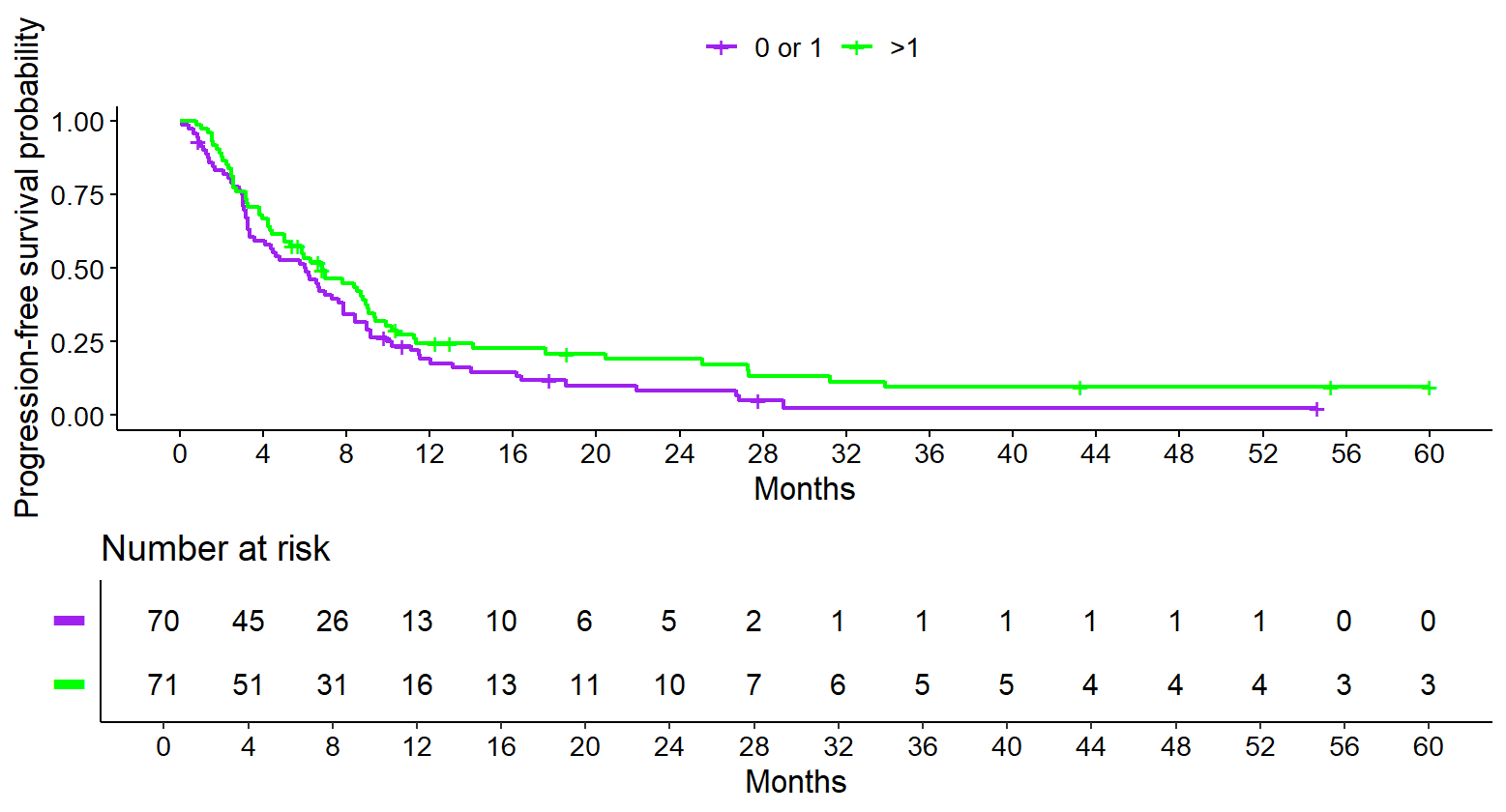


**Figure S2**: Real-world overall survival relative to different subgroups

**Figure S2a**: Real-world overall survival relative to the age


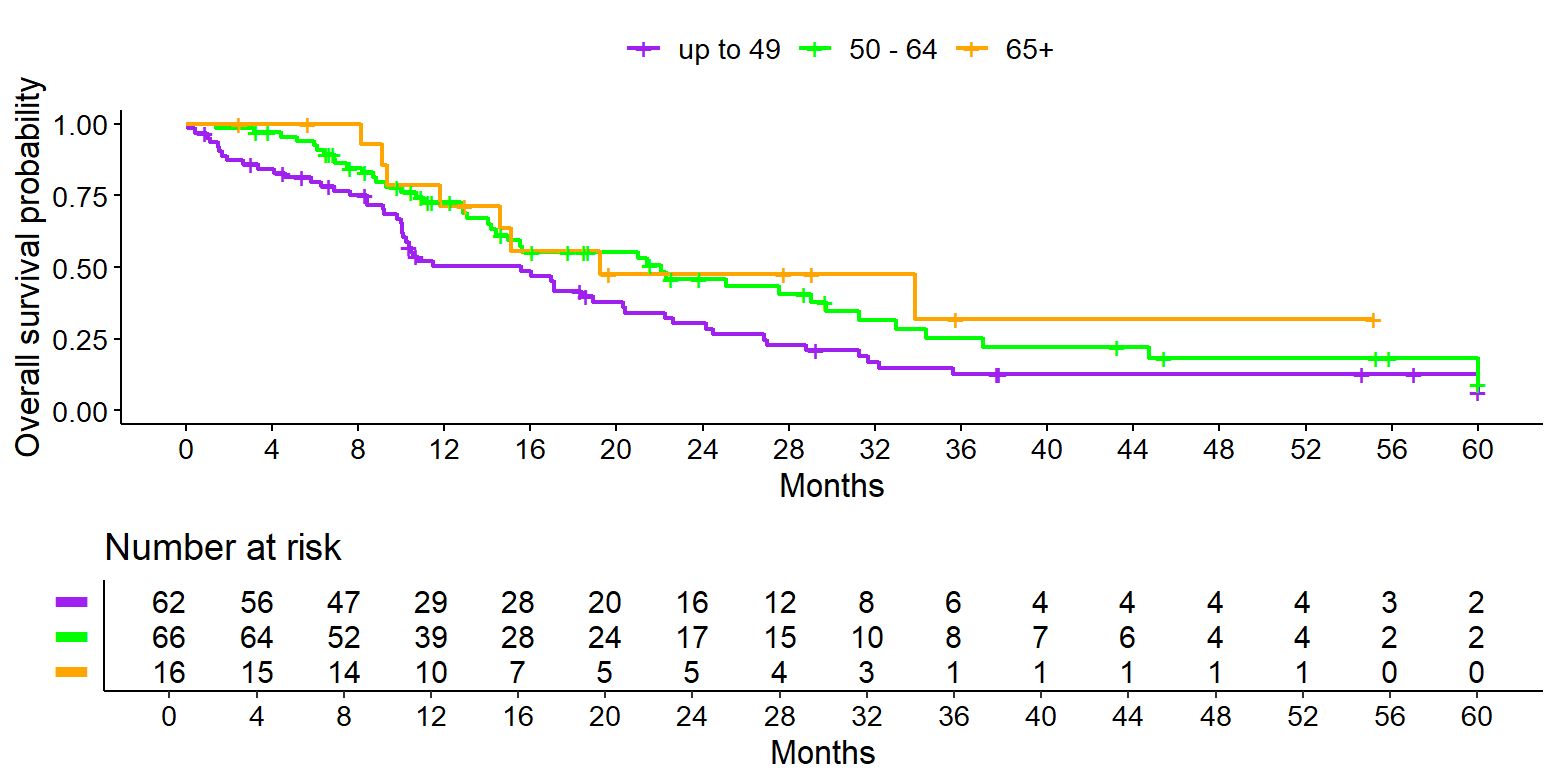


**Figure S2b**: Real-world overall survival relative to the body mass index


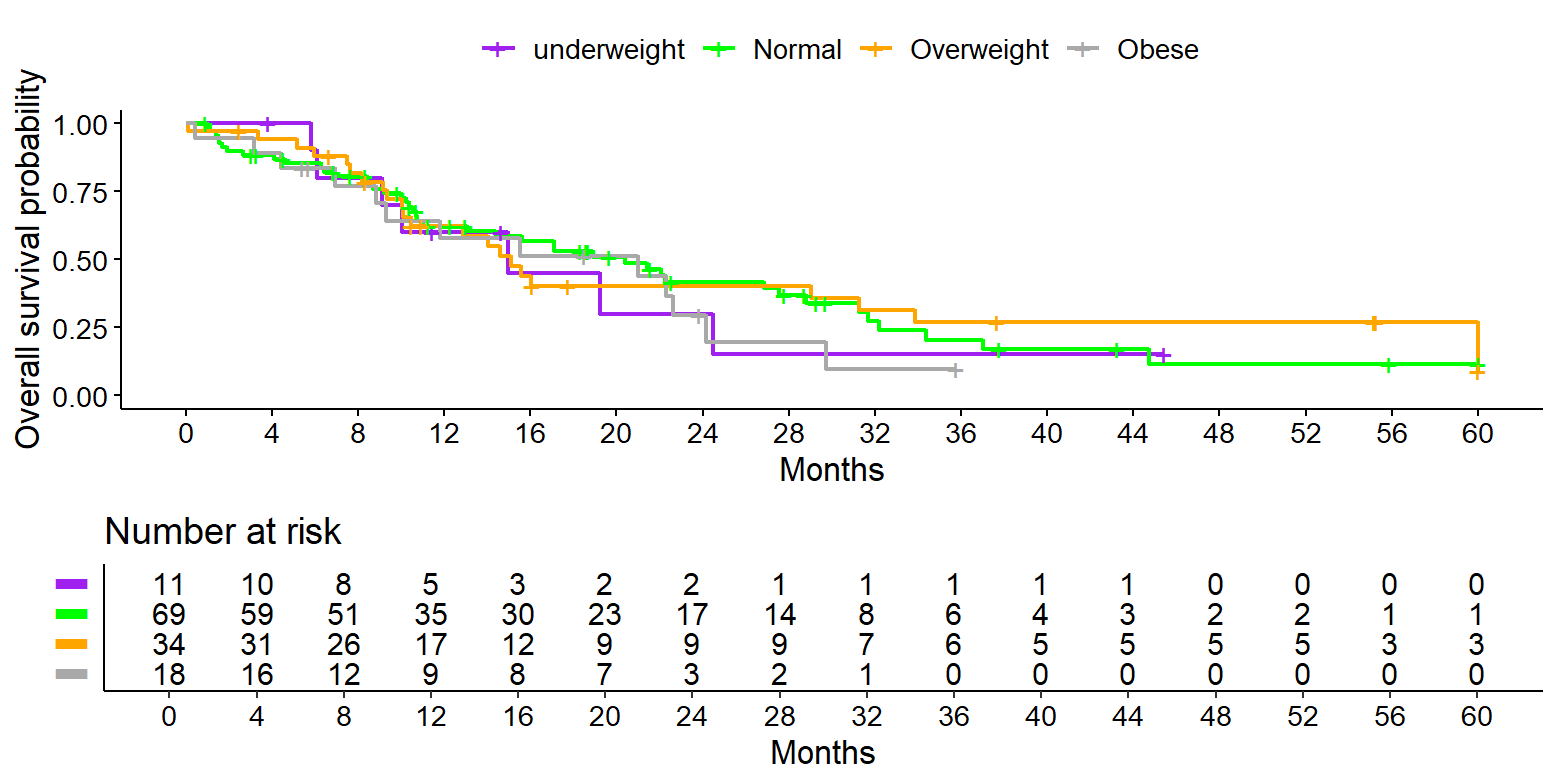


**Figure 2c**: Real-world overall survival relative to the Eastern Cooperative Oncology Group (ECOG) performance status


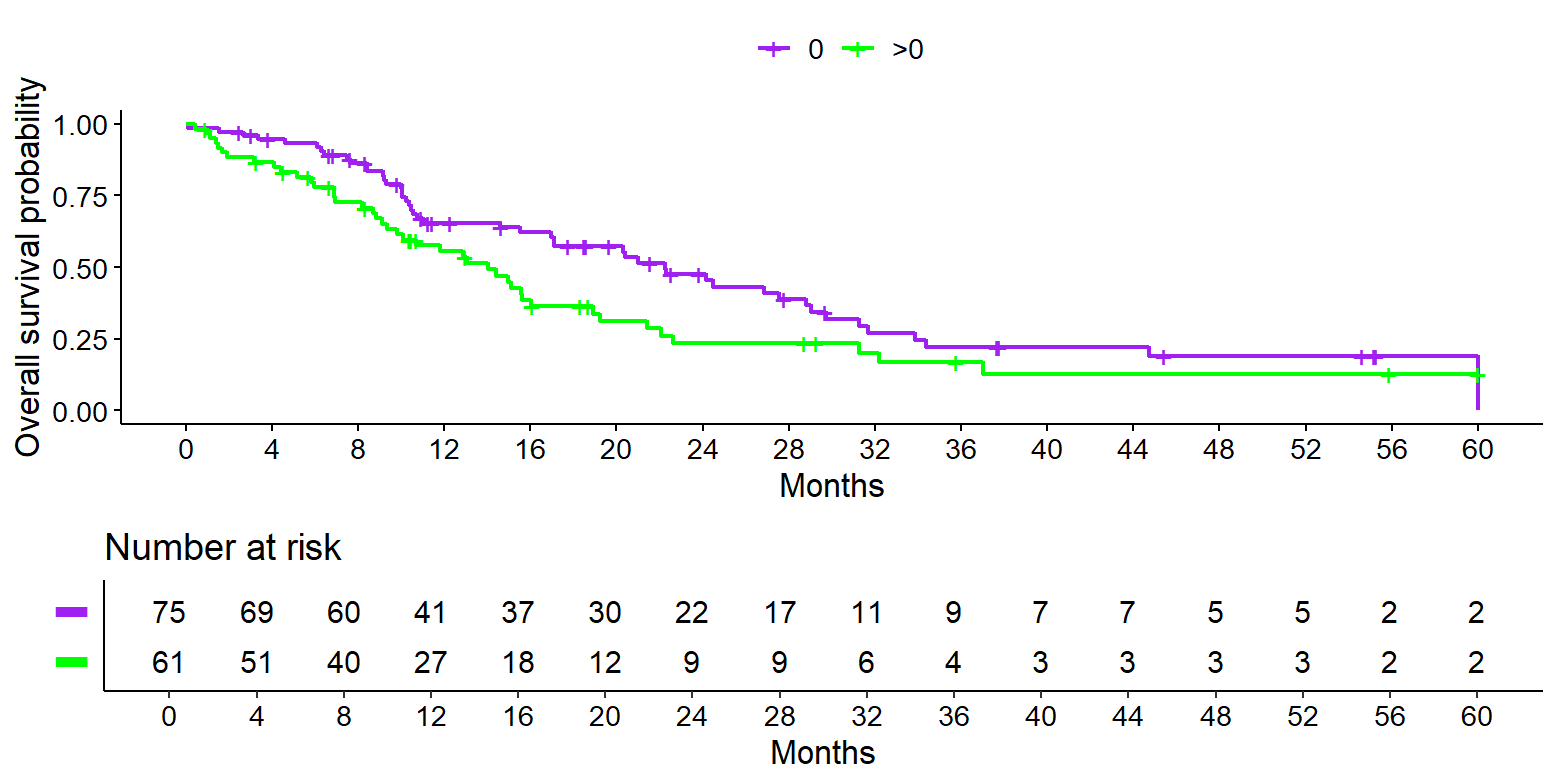


**Figure S2d**: Real-world overall survival relative to the tumor grading


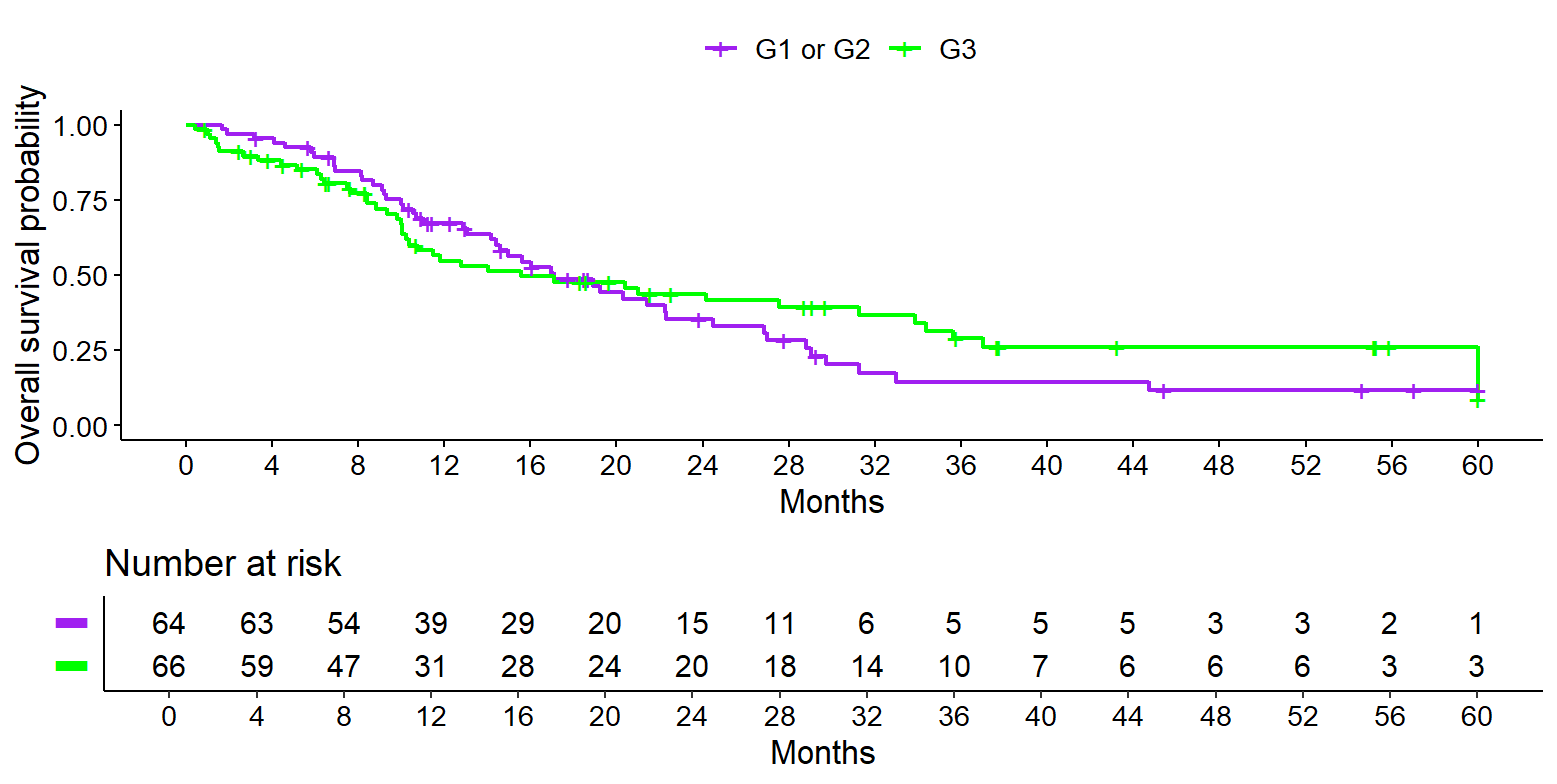


**Figure S2e**: Real-world overall survival relative to the line of therapy


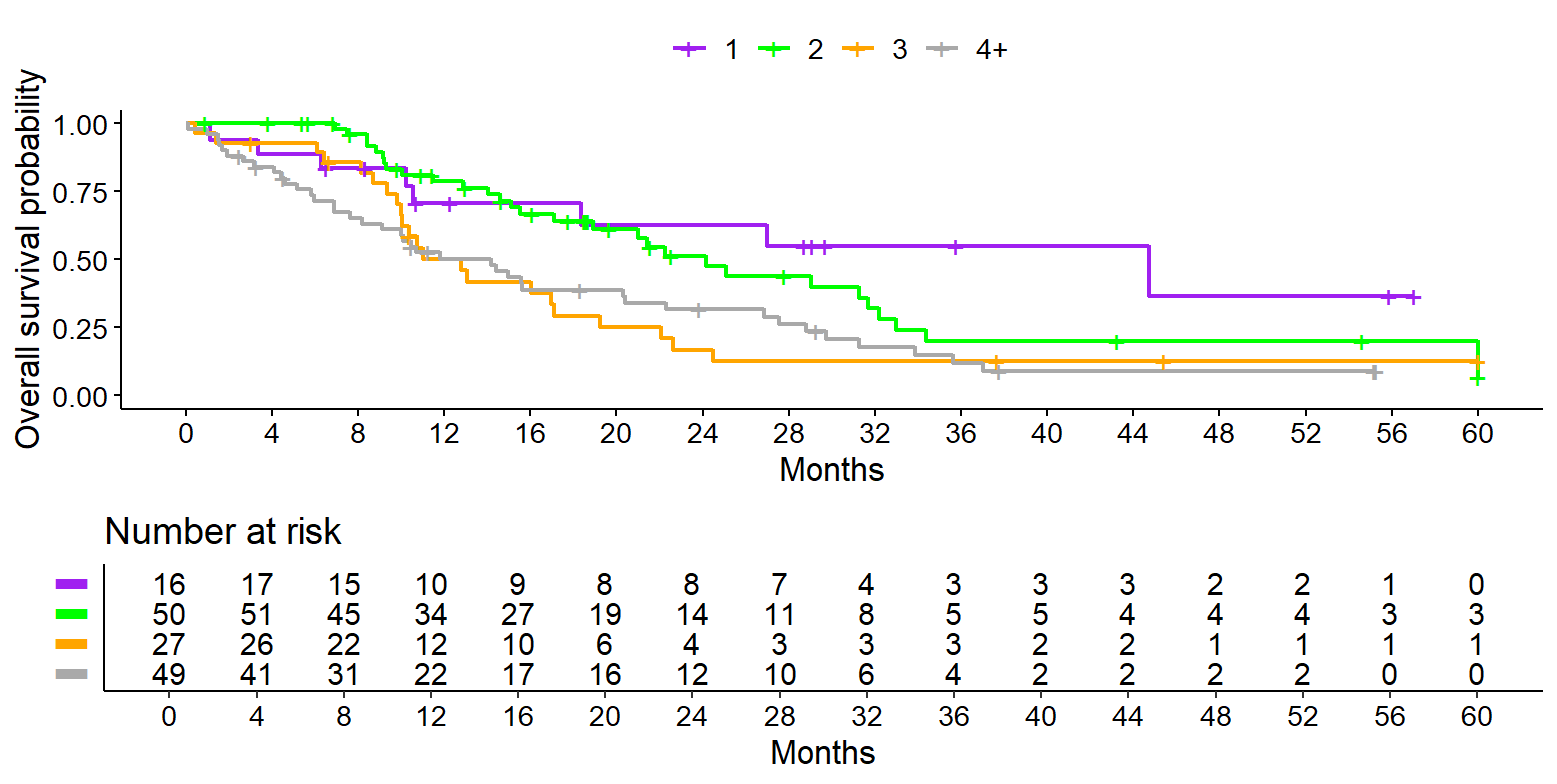


**Figure S2f**: Real-world overall survival relative to metastasis pattern


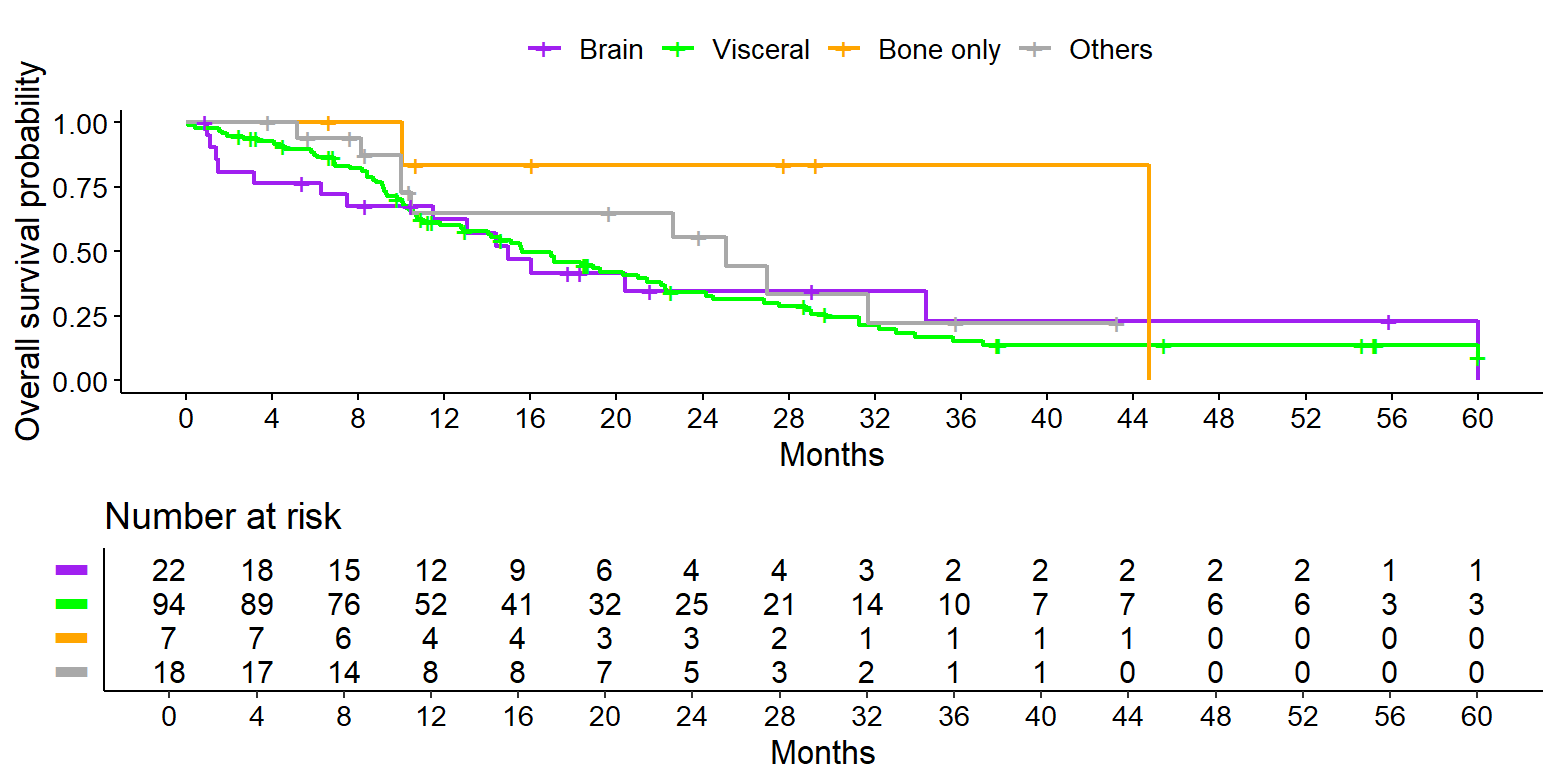


**Figure S2g**: Real-world overall survival relative to metastasis timing (de novo, ≤ 60 months after primary diagnosis, > 60 months after primary diagnosis)


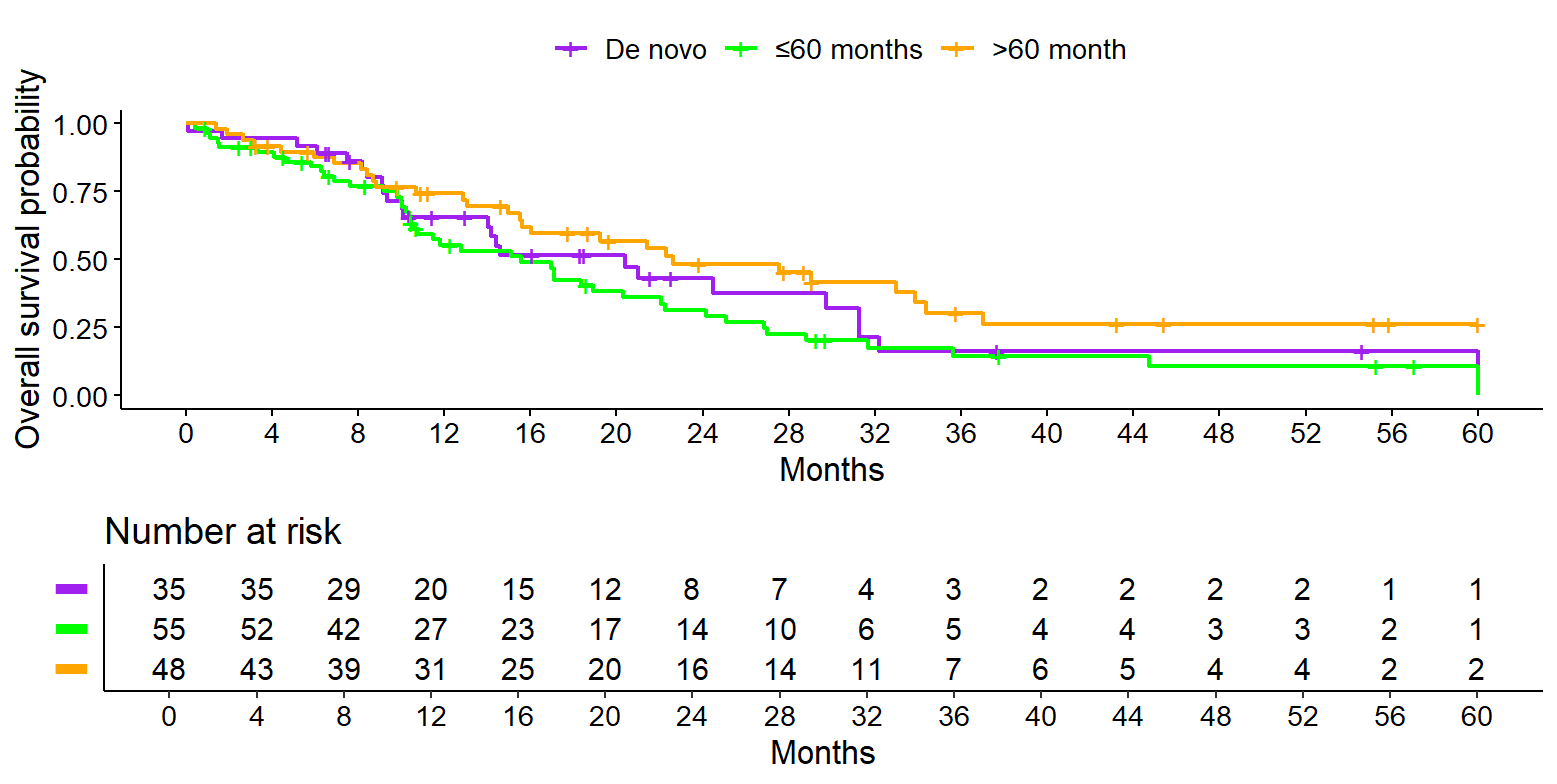


**Figure S2h**: Real-world overall survival relative to the number of concomitant diseases


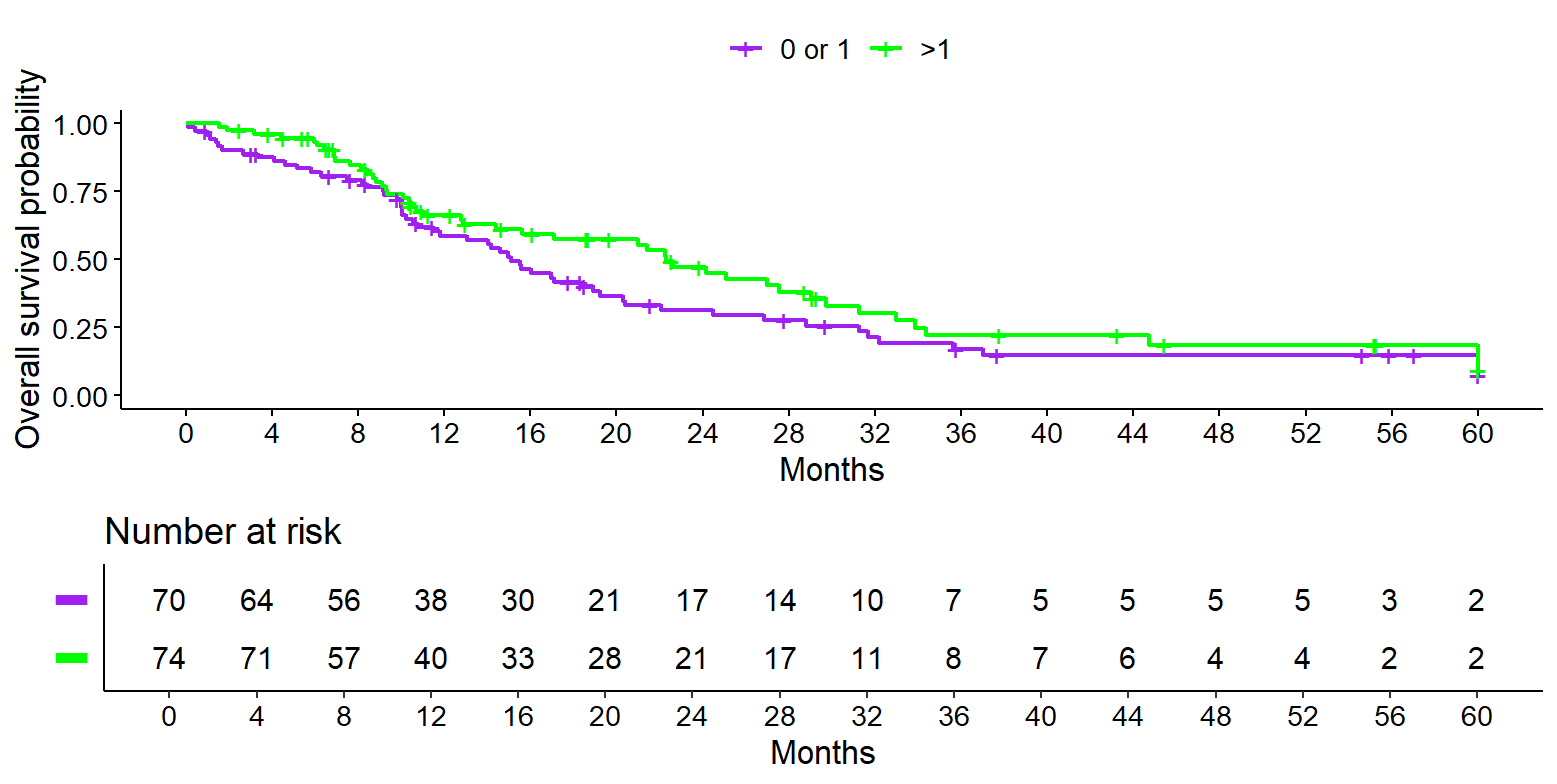


**Figure S3:** Real-world progression-free survival relative to line of therapy and hormone receptor (HR) status

**Figure S3a**: Real-world progression-free survival relative to line of therapy in patients with hormone receptor (HR) positive tumors


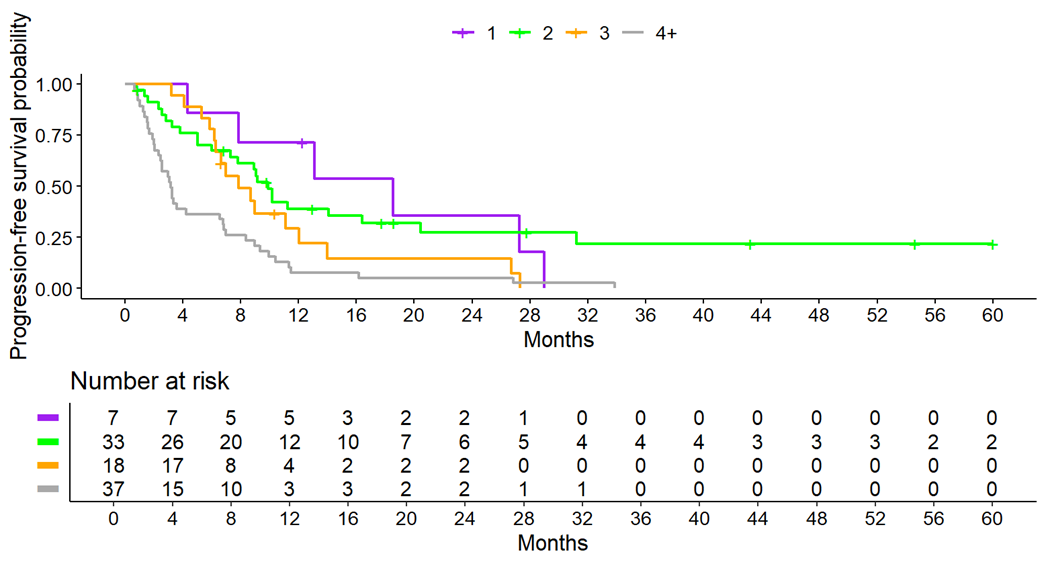


**Figure S3b**: Real-world progression-free survival relative to line of therapy in patients with triple-negative tumors


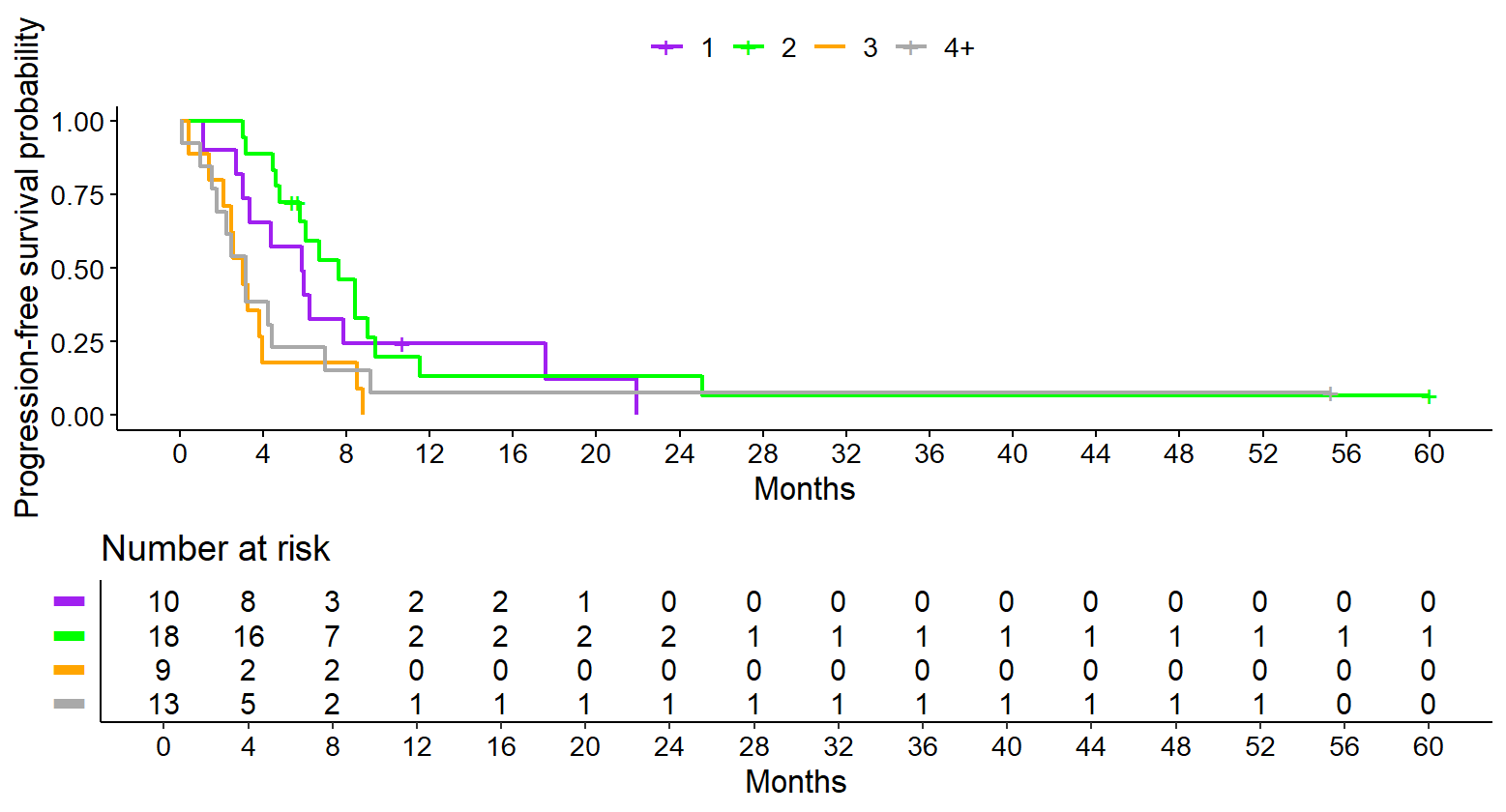


**Figure S4**: Real-world overall survival relative to line of therapy and hormone receptor (HR) status

**Figure S4a**: Real-world overall survival relative to line of therapy in patients with hormone receptor (HR) positive tumors


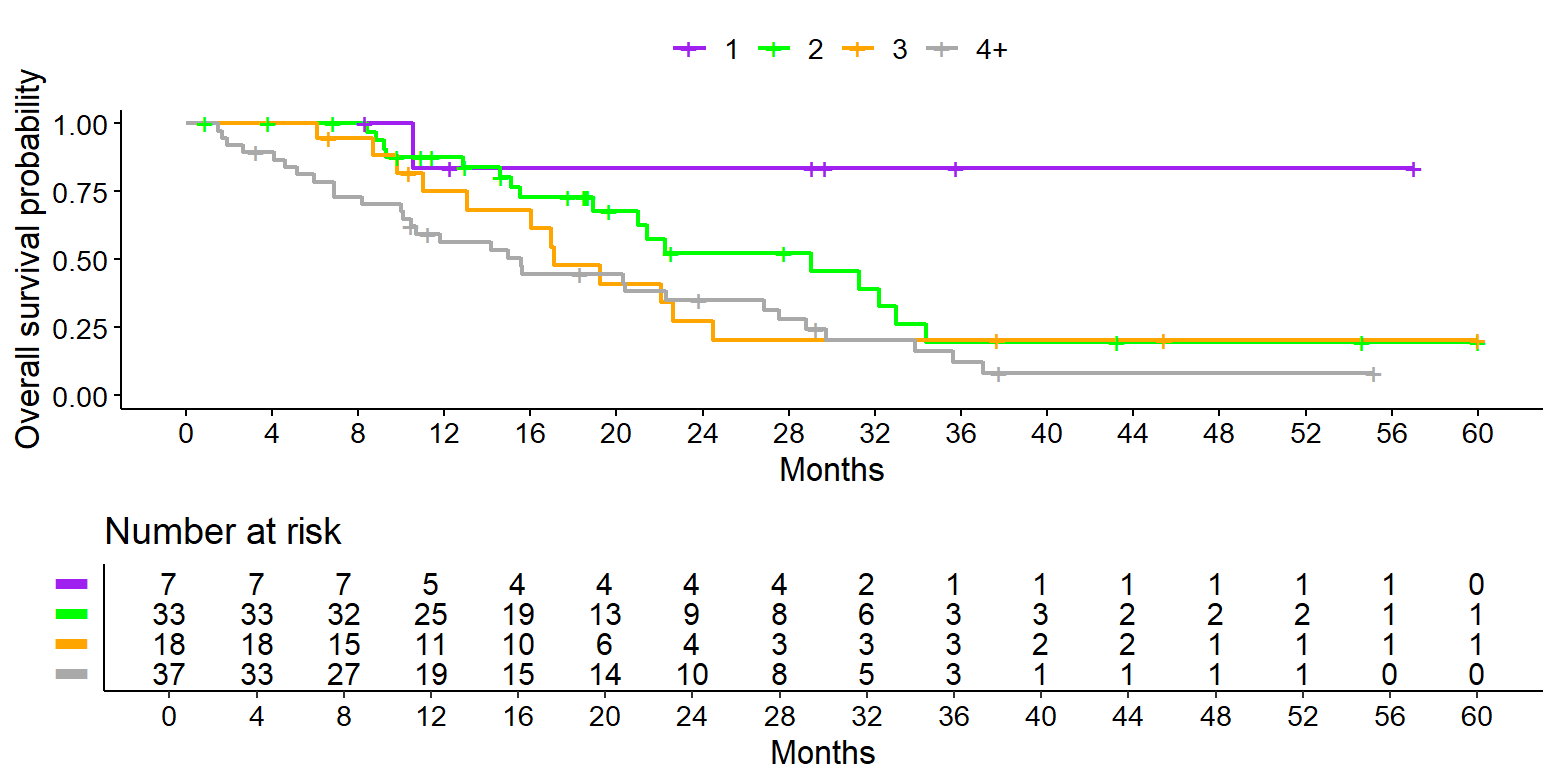


**Figure S4b**: Real-world overall survival relative to line of therapy in patients with hormone receptor triple-negative tumors


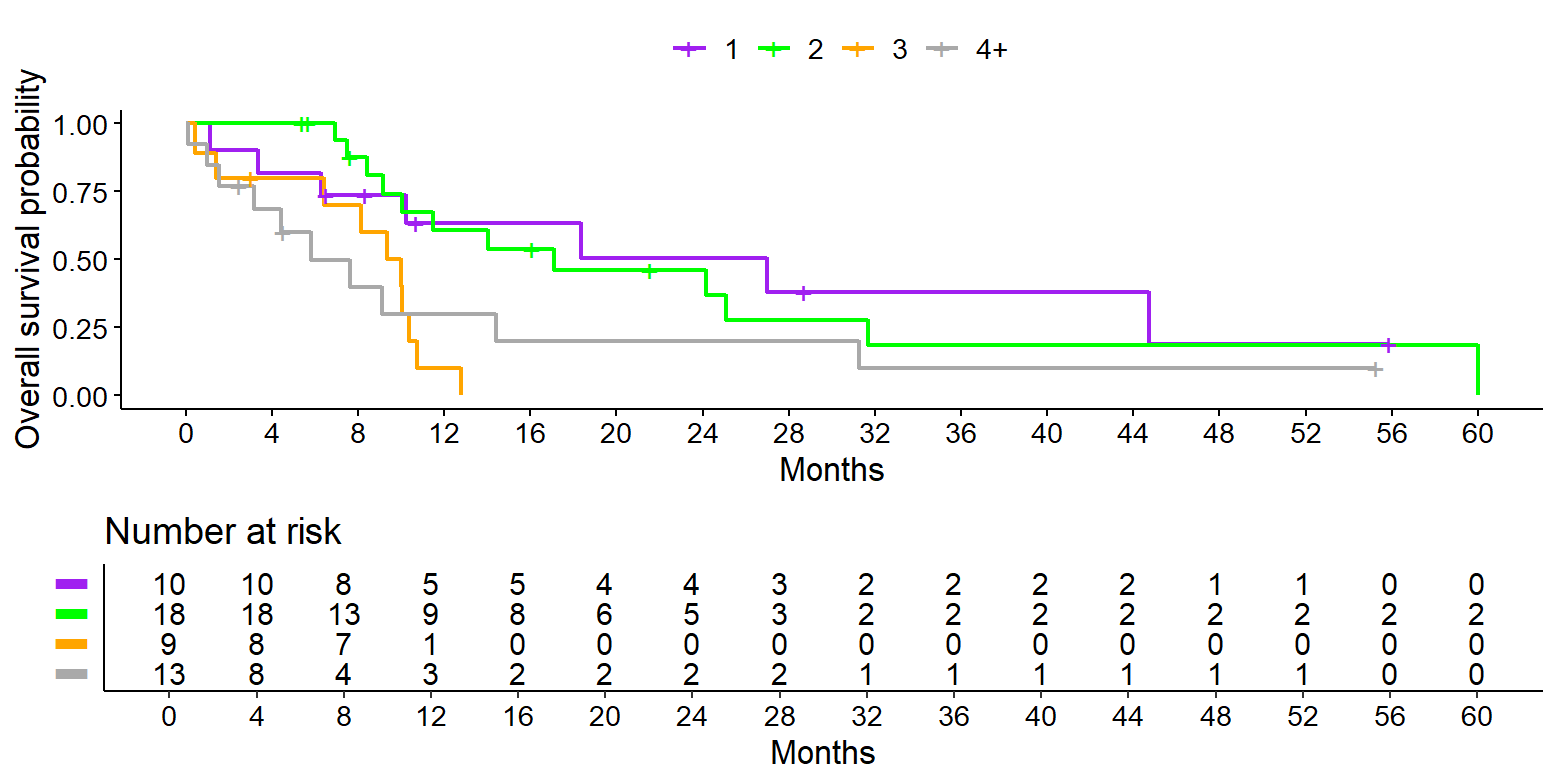

Supplement: Supplementary file 1 — Supplementary information [file 41523_2026_947_MOESM1_ESM.docx]
